# Mild acute biliary pancreatitis: still a surgical disease. A post-hoc analysis of the MANCTRA-1 international study

**DOI:** 10.1007/s00068-024-02748-9

**Published:** 2025-01-17

**Authors:** Stefano Piero Bernardo Cioffi, Andrea Spota, Francesco Virdis, Michele Altomare, Andrea Mingoli, Stefania Cimbanassi, Francesca Laura Nava, Silvana Nardi, Marcello Di Martino, Salomone Di Saverio, Benedetto Ielpo, Francesco Pata, Gianluca Pellino, Massimo Sartelli, Dimitris Damaskos, Federico Coccolini, Adolfo Pisanu, Fausto Catena, Mauro Podda

**Affiliations:** 1 General Surgery Trauma Team, Niguarda Hospital, Piazzale Dell’ospedale Maggiore 3, 20162 Milan, Italy; 2 Department of Surgery, University of Rome Sapienza, Viale del Policlinico 155, 00161 Rome, Italy; 3 Department of Surgical Pathophysiology and Transplant, University of Milan, Milan, Italy; 4 University of Milan, Milan, Italy; 5 San Raffaele University, Milan, Italy; 6 Department of Health Sciences, University of Piemonte Orientale, Novara, Italy; 7 General Surgery Unit Head, AST Ascoli Piceno, Madonna del Soccorso Hospital, San Benedetto del Tronto, Italy; 8 Hepatobiliary Surgery Unit, Hospital del Mar, Barcelona, Spain; 9 Department of Pharmacy, Health and Nutritional Sciences, University of Calabria, Rende, Italy; 10 Colorectal Unit, Vall d’Hebron University Hospital, Universitat Autonoma de Barcelona UAB, Barcelona, Spain; 11 General and Emergency Surgery, Macerata Hospital, Macerata, Italy; 12 General and Emergency Surgery, Royal Infirmary of Edinburgh, Edinburgh, UK; 13 General, Emergency and Trauma Surgery Dept, Pisa University Hospital, Pisa, Italy; 14 Department of Surgical Science, University of Cagliari, Cagliari, Italy; 15 General and Emergency Surgery, Bufalini Hospital, Cesena, Italy

**Keywords:** Acute biliary pancreatitis, Mild, Early cholecystectomy, Recurrence, Readmission, Guidelines

## Abstract

**Background:**

The current standard of care for mild acute biliary pancreatitis (MABP) involves early laparoscopic cholecystectomy (ELC) to reduce the risk of recurrence. The MANCTRA-1 project revealed a knowledge-to-action gap and higher recurrence rates in patients admitted to medical wards, attributable to fewer ELCs being performed. The project estimated a 35% to 70% probability of narrowing this gap by 2025. This study evaluates the safety of suboptimal ELC implementation and identifies risk factors for recurrent acute biliary pancreatitis (RAP) in patients not undergoing ELC after an MABP episode.

**Methods:**

We conducted a post-hoc analysis of the MANCTRA-1 registry, including MABP patients who did not undergo ELC during the index hospitalization, excluding those with related complications. The primary outcome was the 30-day hospital readmission rate due to RAP. We performed multivariable logistic regression to find risk factors associated with the primary outcome.

**Results:**

Between January 2019 and December 2020, 1920, MABP patients from 150 centers were included in the study. The 30-day readmission rate due to RAP was 6%. Multivariable logistic regression found the admission to a medical ward (internal medicine or gastroenterology) (OR = 1.95, p = 0.001) and a positive COVID-19 test (OR = 3.08, p = 0.029) as independent risk factors for RAP.

**Conclusion:**

Our analysis offers valuable insights into the management of MABP, particularly in centers where ELC cannot be fully implemented due to logistical and clinical constraints, worsened by the COVID-19 pandemic. Regardless of the admitting ward, prompt access to surgical care is crucial in reducing the risk of early recurrence, highlighting the need to implement surgical consultation pathways within MABP care bundles.

**Supplementary Information:**

The online version contains supplementary material available at 10.1007/s00068-024-02748-9.

## Introduction

Mild acute biliary pancreatitis is a subset of acute pancreatitis cases, which demands careful consideration of the most effective treatment strategies. Over the years, the management of MABP has evolved, and a key aspect that has gained increasing attention is the timing of cholecystectomy.

Early laparoscopic cholecystectomy, compared to less invasive but not definitive approaches, has been demonstrated to be critical in preventing recurrent acute biliary diseases also for acute cholecystitis, as shown in the CHOCOLATE trial [1].

Historically, in MABP the conventional approach advocated for delayed cholecystectomy following the resolution of the acute inflammatory episode to reduce the risk of surgical complications associated with local inflammation. However, a growing body of evidence has challenged this paradigm, highlighting the benefits of ELC in the management of MABP. Early surgical intervention holds the promise of not only preventing RAP but also mitigating the potential for gallstone-related complications, such as common bile duct obstruction from choledocholithiasis [2–7].

The timing of cholecystectomy, once considered a cautious undertaking, emerges as a potentially transformative intervention, redefining the landscape of MABP management [8, 9].

The MANCTRA-1 project [9], an international multi-institutional audit, explored the current knowledge-to-action (KTA) gap in acute pancreatitis, disclosing how, despite the evidence available, healthcare providers still fail to provide the recommended standard of care to patients with MABP. In the general population of the study, the adherence to ELC implementation was 29%, with the lowest rates in patients admitted to medical wards and higher rates of RAP in those specifically admitted to gastroenterology units.

The final effort of the project provided the most accurate and rigorous clinical bundle available for acute biliary pancreatitis with seven key elements, recommending the implementation of ELC. The human and AI predictions for the accomplishment of optimal care on the implementation of the ELC, estimate a probability ranging from 34 to 70% of narrowing the KTA by 2025 [10]. Thus, a non-negligible percentage of health-care providers may not be able to implement evidence-based practices in the future.

The theoretical framework behind this study is that the lack of awareness of guidelines is unlikely to be the only contributing factor to poor compliance. Limited hospital resources, long waiting lists, poor empowerment, and common beliefs can be the main contributors to the KTA. Furthermore, we hypothesized that there could still be space to improve patients’ outcomes when guidelines cannot be implemented in patients with MAPB and no contraindications to ELC.

This study aims to find modifiable factors associated with the 30-day recurrence rate of ABP when ELC was shown as feasible but still not performed in MABP.. The real-life insights provided by this post-hoc analysis could hopefully integrate the care bundles on acute biliary pancreatitis published by the MANCTRA-1 steering group [10].

## Methods

### Study design

The primary outcome of the study was the rate of 30-day RAP requiring hospitalization while waiting for interval cholecystectomy, in patients with MABP not operated on during the index hospitalization, despite the absence of contraindication to surgery.

The present study is a post hoc examination of the MANCTRA-1 study dataset, which took place across 150 centers spanning Europe, Asia, Africa, South America, and Oceania [9, 11].

Ethical approval for the MANCTRA-1 study and its subsequent post hoc analyses was obtained from the Institutional Review Board of the University of Cagliari (Italy) (PROT. P.G./ 2021/5410–31/03/2021) and local boards of the participating centers. This research adhered to the principles outlined in the Declaration of Helsinki and was developed and presented following the guidelines of Strengthening the Reporting of Observational Studies in Epidemiology (STROBE), ClinicalTrials. Gov NCT04747990 [9, 12].

### Selection criteria

A retrospective analysis was conducted including all consecutive patients hospitalized between January 2019 and December 2020, with a diagnosis of MABP based on the Revised Atlanta Classification not undergoing ELC during the index hospitalization [13].

Patients with MABP and common duct biliary stones, without cholangitis, undergoing uneventful endoscopic treatment were included in the analysis, and no contraindication to undergo cholecystectomy after ERCP.

Patients were excluded from this analysis if they experienced any clinical event contraindicating the execution of an ELC, such as complications from MABP, clinical evolutions of MABP, or underwent interventional procedures to treat MABP-related complications.

### Variables of interest

Variables of interest were considered from the MANCTRA-1 dataset, considering the following data: baseline, medical and surgical history, clinical data on admission, the destination of admission (Hepato-Pancreatico-Biliary surgery ward, General Surgery ward, Internal Medicine ward, Gastroenterology ward), medical and surgical management.

### Statistical analysis

The baseline characteristics of the study population were presented using absolute numbers and relative frequency measurements for qualitative variables, while quantitative variables were expressed as mean and standard deviation (SD) or median and standard error (SE)/Interquartile Range (IQR).

A dummy variable was created for the variable “admitting ward,” enclosing patients admitted to HPB or general surgery ward in the variable “surgical ward” and those admitted to internal medicine or gastroenterology ward in the variable “medical ward”.

The X2 test or Fisher's exact test, as appropriate, was employed to evaluate differences between groups for qualitative variables. To compare patients who experienced RAP while awaiting interval cholecystectomy with those who did not, the student t-test was used for variables with a parametric distribution, and the Mann–Whitney U test was used for those with a non-parametric distribution.

A multivariable logistic regression was performed to assess the relation between 30-day Hospital readmission due to RAP and the explanatory variables, selected among the significant ones in the univariate analysis, with clinical significance. Significant variables from the univariable analysis were not included in the multivariable model if the percentage of missing data exceeded 15%, avoiding to utilize missing data imputation to keep the fidelity with real-world information. Data were checked for multicollinearity with the Belsley-Kuh-Welsch technique. Heteroskedasticity and normality of residuals were assessed respectively by the White test and the Shapiro–Wilk test. A p-value of 0.05 was considered statistically significant. Missing data were imputed as the mean for the numeric variables and the most frequent modality for discrete variables. Statistical analysis was performed with EasyMedStat (version 3.31; www.easymedstat.com).

## Results

From 01/01/2019 and 31/12/2020, 5275 patients from 150 centres worldwide were included in the MANCTRA-1 trial. For this post-hoc analysis we applied the above-mentioned inclusion and exclusion criteria and 1920 patients with MABP and no disease-related complications, not undergoing ELC during index hospitalization, were finally considered. The rate of RAP in the selected population was 6.25%, 120 patients.

**Fig. 1:**
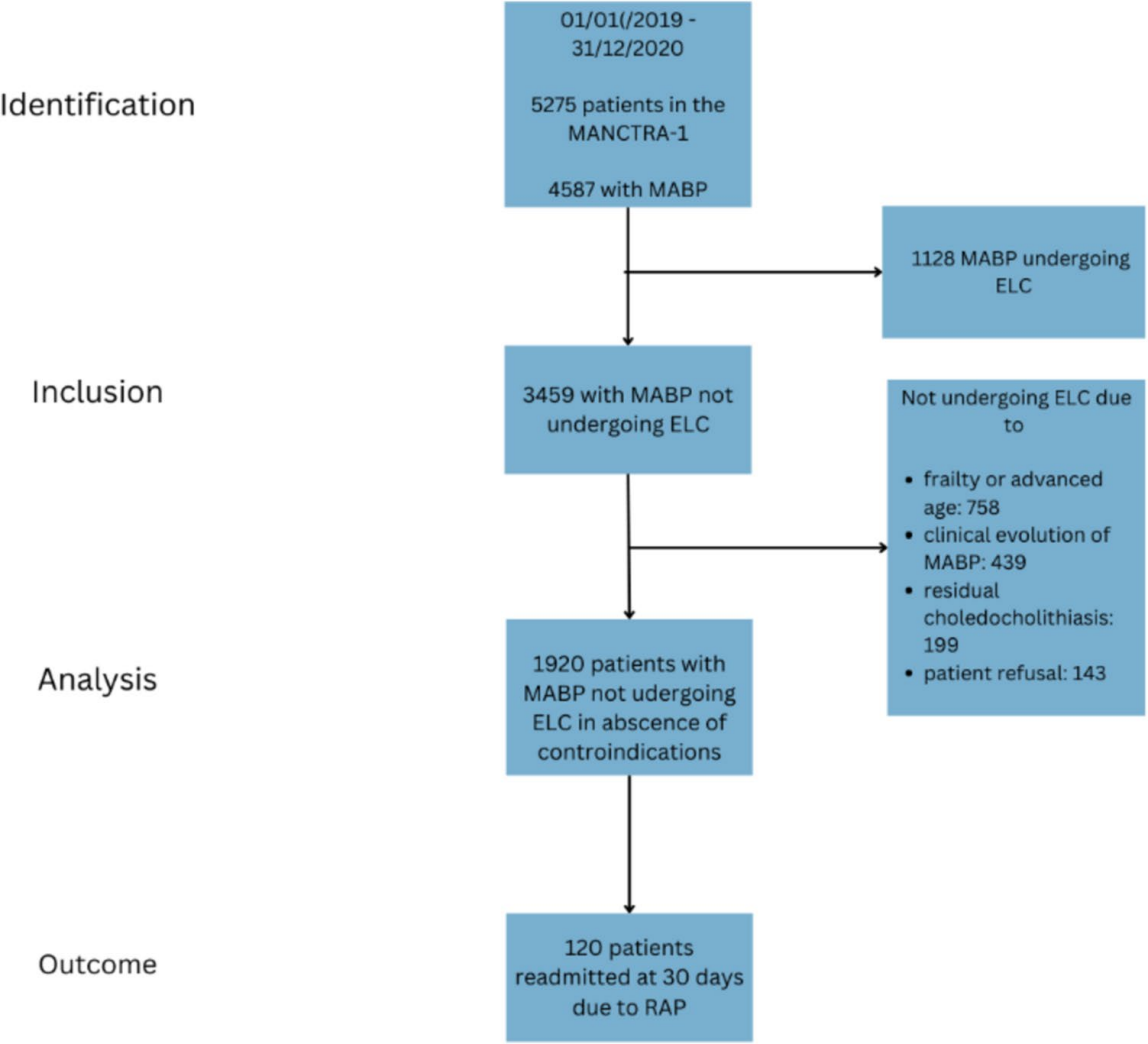
The flowchart of patients enrolled in this post-hoc study is reported. Specific reasons for patients’ exclusion are reported. MABP, Mild Acute Biliary Pancreatitis. ELC, Early Laparoscopic Cholecystectomy. RAP, recurrent Acute Pancreatitis

The flowchart of patients’ enrolment is reported in Fig. 1.

The results of the univariable analysis, reported in the supplementary materials S3, showed the differences between patients readmitted due to RAP at 30 days and those who did not recur.

Specifically, RAP rates were not lower in patients undergoing ERCP for choledocholithiasis or in those without previous episodes of ABP. Younger age, positive COVID testing, admission to a medical ward, lower Body Mass Index, higher values of White Blood Cells, Ranson’s criteria, amylase, and lactates were significantly associated with higher recurrence rates.

Ranson’s criteria, amylase, lactate, and BMI, despite being significantly associated with RAP were not included in the model due to missing data.

White blood cells on admission (cells/mm3) (OR = 1.07, [1.02; 1.11], p = 0.002), being admitted to a medical ward (OR = 1.95, [1.3; 2.91], p = 0.0012), and having a Covid-19 positive testing on admission (OR = 3.08, [1.12; 8.5], p = 0.0294) were associated with higher rates of 30-day hospital readmission due to RAP at the multivariable analysis (Table 1).

**Table 1:** Results of the Multivariable Logistic Regression model

variable	modality	Adjusted odds ratio	p-value
Intercept		0.0578 [0.0256;0.131]	** < 0.001**
Patient age		0.984 [0.974;0.994]	**0.002**
Covid-19 status on admission	Untested (reference)		
	Negative	0.691 [0.467;1.02]	0.064
	Positive	3.08 [1.12;8.5]	**0.029**
Admission to a medical ward		1.95 [1.3;2.91]	**0.001**
WBC on admission (cells/mm3)		1.07 [1.02;1.11]	**0.002**

Significant variables are reported in bold

*WBC* white blood cells

**Fig. 2:**
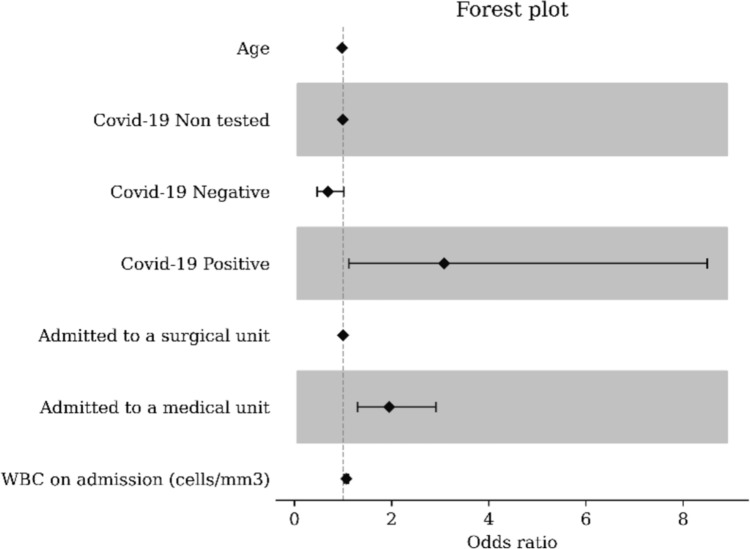
The significant impact of positive Covid-19 testing and the admission to a medical ward on 30-day recurrence, when ELC is not implemented in MABP, is depicted in the multivariable logistic regression model

In Fig. 2 the forest plot of the multivariable logistic regression is depicted.

## Discussion

Following the results of the MANCTRA-1 audit [9], we investigated modifiable risk factors for 30-day hospital readmission due to recurrent acute pancreatitis (RAP) in a selected population of MABP patients not undergoing ELC without clinical contraindications. Our hypothesis was that centers unable to implement ELC in suitable MABP cases could still improve patient outcomes through alternative strategies, creating a "safety zone" between evidence-based and non-evidence-based practice and integrating published care bundles [10]^.^

In our analysis, 6.25% (120/1920) of MABP patients not undergoing ELC were readmitted within 30 days due to RAP. This finding aligns with broader literature showing RAP rates ranging from 8.99–20% [3, 14–17], with biliary-related RAP specifically reported at approximately 12% in recent meta-analyses [18].

The rate reported in our study, derived from a population with no theoretical contraindications to ELC, is comparable to the 6.8% reported in the original MANCTRA-1 study [9]. In this post-hoc study we applied strict exclusion criteria to analyze a selected population of patients in which no clinical contraindication or patient-related constraint could theoretically hamper the possibility of performing ELC.

Two significant predictors of 30-day RAP readmission were disclosed: hospitalization in medical wards and positive COVID-19 testing on admission. Additionally, ERCP in patients with common bile duct stones showed no protective effect against RAP when ELC was not subsequently performed.

Our definition of RAP focused on 30-day readmission rates to specifically examine the burden of preventable hospitalizations related to poor ELC implementation. This approach differs from traditional definitions that consider RAP as separate attacks with complete recovery periods of at least three months, as symptoms within three months post-attack are often viewed as complications rather than true recurrence [15, 19–21].

Our analysis confirmed and reinforced the results of MANCTRA-1 original paper [9] showing that, in a selected population with MABP where guidelines on ELC were not followed, the recurrence rates are higher in patients admitted to medical wards. This sub-analysis showed that, when logistical and organizational constraints hamper evidence-based practice implementation, clinical pathways may still be improved to enhance patients’ safety.

The admitting specialty has already been studied in the past as a discriminating factor in the management of biliary pancreatitis. Mohy-ud-din et al. [22] have reported management differences between medicine and surgery services. While both surgical and medical practice guidelines recommend cholecystectomy for preventing recurrent biliary pancreatitis, surgical societies strongly advocate for index admission cholecystectomy, whereas medical guidelines vary in their approach [23].

Variation exists even among surgical specialties, with hepatobiliary surgeons showing better guideline compliance [16]. British audit data showed also that waiting times could be influenced by possible lower surgical confidence among non-HPB surgeons [24]. The MANCTRA-1 audit revealed that general surgery departments may often provide more timely definitive treatment due to better access to emergency theatre slots [9].

The advantages of dedicated operative pathways for acute surgical patients are well documented [25, 26]. Since 1998, guidelines have emphasized that hospitals receiving acute pancreatitis admissions should have specialist services or coordinated referral systems [6, 27]

The reality is still discrepant from ideal and theoretical clinical pathways. The ORSA study, surveying 147 hospitals worldwide, revealed that most centers lack dedicated emergency operating rooms, causing delays in both elective and acute cases [28].

The COVID-19 risk factor must be contextualized within the pandemic's impact on surgical practices. During the initial outbreak, uncertainty about optimal timing for cholecystectomies in COVID-positive patients, combined with reduced operative activities and shifted priorities, affected patient management [28–31, 31–34]. However, delaying surgery due to the COVID-19 infection may be considered an indirect demonstration of the effect of timing on the risk of RAP, highlighting the importance of an ELC.

The univariable analysis also offered an interesting insight highlighting that performing an ERCP in the presence of common bile duct stone, does not seem to mitigate RAP rates if ELC is not subsequently performed. Endoscopy still plays a critical role, but not sufficient to shift the focus from dedicated surgical pathways [35, 36].

This study has several limitations. Its retrospective nature prevents the analysis of variables that other studies have shown related to RAP (i.e., smoking, local complications) [15, 18, 18]. We included several hospitals worldwide, in which protocols are often different, with high heterogeneity in the medical and surgical management of ABP patients [15]. The multicentric nature of the MANCTRA-1 audit reflects the current real-world practice, increasing the external validity of our results, hampered by the high rate of European hospitals involved. Indeed, these results may be impactful for healthcare providers managing MABP in Western Countries.

Finally, our results should be interpreted considering the same clinical and logistical constraints limiting the implementation of evidence-based practices, and finding a safety zone in which patients with mild acute biliary pancreatitis can be managed. The focus should be shifted to creating and improving dedicated pathways for MABP patients, guaranteeing prompt access to surgical consultation, regardless of the admitting ward.

**Fig. 3:**
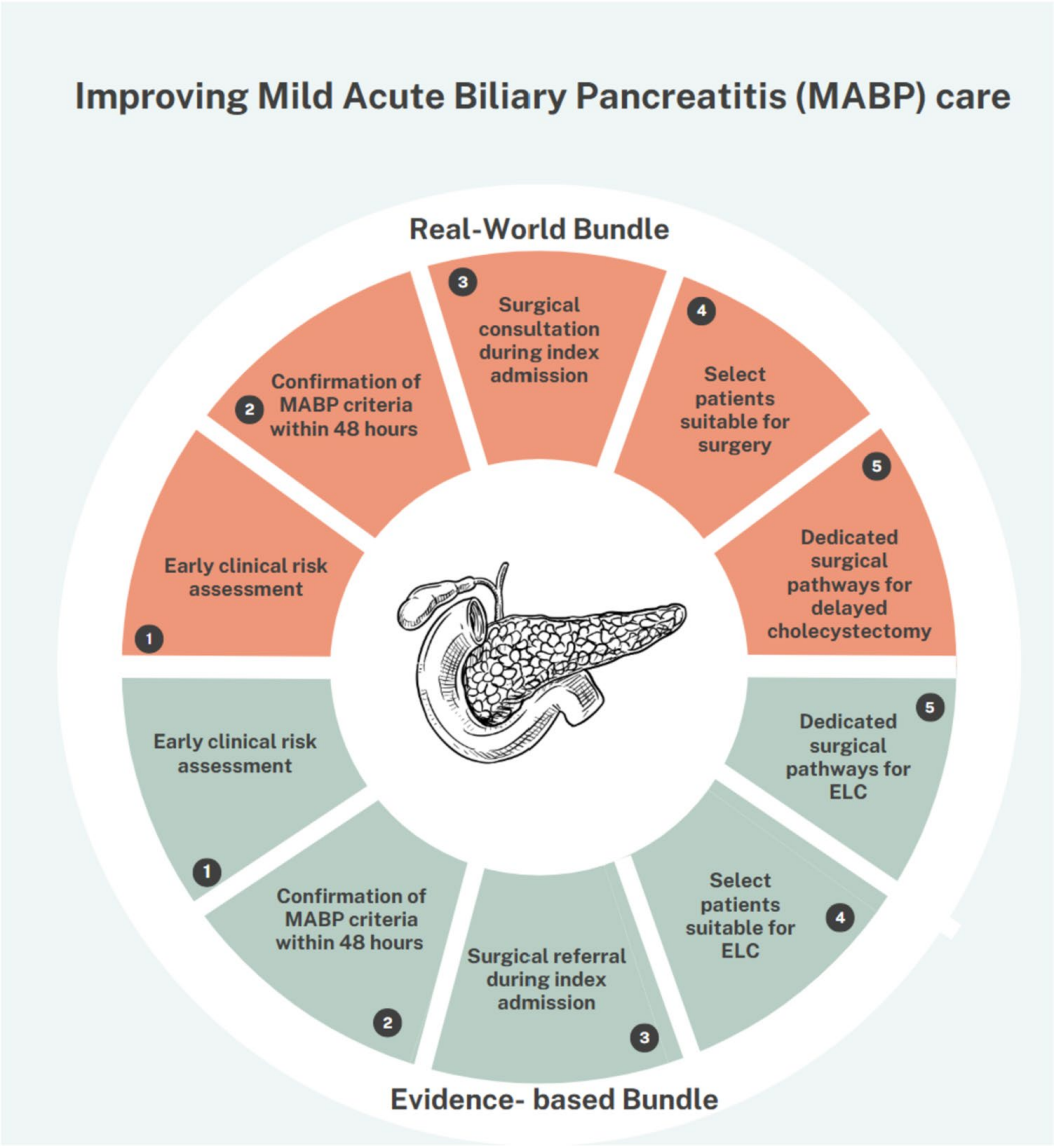
In the upper, light-red, half of the circle, a real-world bundle is depicted to improve Mild Acute Biliary Pancreatitis (MABP) care, informing healthcare providers on preventive strategies to mitigate recurrent pancreatitis when early laparoscopic cholecystectomy cannot be implemented. The lower, light green, half of the circle shows an ideal pathway to implement MABP management

We drafted an integrative bundle of care informing healthcare professionals on alternative strategies to improve MABP patients’ outcomes beyond clinical guidelines. The bundle is shown in Fig. 3.

## Conclusion

Despite the consistency of current recommendations, the knowledge-to-action gap in ELC implementation persists widely due to logistical obstacles, historical assumptions, and poor awareness of evidence. We showed that, when guidelines are not implemented on ELC, admission to a non-surgical ward may further limit the possibility of optimizing MABP patients' outcomes. Future research should also focus on investigating the surgeon and environment-related barriers, affecting compliance with the implementation of ELC.

## Supplementary Information

Below is the link to the electronic supplementary material.

Supplementary file 1

Supplementary file 2

Supplementary file 3

## Data Availability

The data underlying this article will be shared on reasonable request to the corresponding author.
