# Supplementary material for: Mild acute biliary pancreatitis: still a surgical disease. A post-hoc analysis of the MANCTRA-1 international study
Source: Eur J Trauma Emerg Surg. 2025 Jan 17;51(1):24. doi: 10.1007/s00068-024-02748-9 (PMC11742350; doi:10.1007/s00068-024-02748-9)
Supplement: Supplementary file 1 [file 68_2024_2748_MOESM1_ESM.docx]

**MANCTRA-1 Collaborative Group**

Mauro Podda, Chiara Gerardi, Stavros Gourgiotis, Gaetano Poillucci, Kumar Jayant, Cristiana Riboni, Alessio Giordano, Luca Ferrario, Francesco Pata, Gianluca Pellino, Salomone Di Saverio, Benedetto Ielpo, Federico Coccolini, Fausto Catena, Adolfo Pisanu, Vanni Agnoletti, Yoram Kruger, Daniela Pacella, Francesco Virdis, Dimitrios Damaskos, Damian Mole, Belinda De Simone, Ferdinando Agresta, Massimo Sartelli, Ari Leppaniemi, Mikel Prieto Calvo, Michael Wilson, Fiammetta Soggiu, Alaa Hamdan, Carlos Augusto Gomes, Gustavo Fraga, Argyrios Ioannidis, Zaza Demetrashvili, Saaz Sahani, Lovenish Bains, Almu'atasim Khamees, Hazim Ababneh, Osama Aljaiuossi, Samuel Pimentel, Ikhwan Sani Mohamad, Ahmad Ramzi Yusoff, Narcis Octavian Zarnescu, Valentin Calu, Andrey Litvin, Dusan Lesko, Ahmed Elmehrath, Mohamedraed Elshami, Martin de Santibañes, Justin Gundara, Kamel Alawadhi, Rashid Lui, Alexander Julianov, Sergio Ralon, Ibrahim-Umar Garzali, Gustavo M. Machain, Ibabe Villalabeitia, Darwin Artidoro Quispe-Cruz, Abigail Cheska C. Orantia, Maciej Walędziak, Tiago Correia de Sá, Syed Muhammad Ali, Bojan Kovacevic, Colin Noel, Haidar M. Abdalah, Ali Kchaou, Arda Isik, Luca Ansaloni, Walter Biffl, Mario Guerrieri, Alberto Sartori, Manuel Abradelo, Giuseppe Nigri, Nicola Di Lorenzo, Andrea Mingoli, Massimo Chiarugi, Juliana Di Menno Stavron, Oscar Mazza, José Ignacio Valenzuela, Diana Alejandra Pantoja Pachajoa, Fernando Andrés Alvarez, Julian Ezequiel Liaño, Joan Tefay, Abdulrahman Alshaikh, Layla Hasan, Felipe Couto Gomes, Thiago R. A. Calderan, Elcio S. Hirano, Dragomir Dardanov, Alexander Julianov, Azize Saroglu, Boyko Atanasov, Nikolay Belev, Nikola Kovachev, Shannon Melissa Chan, Hon-Ting Lok, Salcedo, Diana Robayo, María Alejandra Triviño, Jan Manak, Saaz Sahani, Jorann de Araujo, Ananya Sethi, Ahmed Awad, Merihan Elbadawy, Ahmed Farid, Asmaa Hanafy, Ahmed Nafea, Sherief-Ghozy, Alzhraa Salah – Abbas, Wafaa Abdelsalam, Sameh Emile, Ahmed Elfallal, Hossam Elfeki, Hosam Elghadban, Ashraf Shoma, Mohamed Shetiwy, Mohamed Elbahnasawy, Salem- Mohamed, Emad Fawzi Hamed, Usama Ahmed Khalil, Nikolaos Koronakis, Elie Chouillard, Andrew Gumbs, Andrea Police, Andrea Mabilia, Kakhi Khutsishvili, AnanoTvaladze, Orestis Ioannidis, Elissavet Anestiadou, Lydia Loutzidou, Konstantinis Konstantinidis, Sofia Konstantinidou, Dimitrios Manatakis, Vasileios Acheimastos, Nikolaos Tasis, Nikolaos Michalopoulos, Panagiotis Kokoropoulos, Maria Papadoliopoulou, Maria Sotiropoulou, Stylianos Kapiris, Panagiotis Metaxas, Ioannis Tsouknidas, Despoina Kefili, George Petrakis, Eirini Synekidou, Konstantinos Dakis, Eirini Alexandridou, Aristeidis Papadopoulos, Christos Chouliaras, Odysseas Mouzakis, Francesk Mulita, Ioannis Maroulis, Michail Vailas, Tania Triantafyllou, Dimitrios Theodorou, Eftychios Lostoridis, Eleni-Aikaterini Nagorni, Paraskevi Tourountzi, Efstratia Baili, Alexandros Charalabopoulos, Theodore Liakakos, Dimitrios Schizas, Alexandros Kozadinos, Athanasios Syllaios, Nikolaos Machairas, Stylianos Kykalos, Paraskevas Stamopoulos, Spiros Delis, Christos Farazi-Chongouki, Evangelos Kalaitzakis, Miltiadis Giannarakis, Konstantinos Lasithiotakis, Giorgia Petra, Evangelos Kalaitzakis, Amit Gupta, Noushif Medappil, Vijayanand Muthukrishnan, Jubin Kamar, Pawan Lal, Rajendra Agarwal, Matteo Magnoli, Paolo Aonzo, Alberto Serventi, Antonio Giuliani, Pierpaolo Di Lascio, Margherita Pinto, Carlo Bergamini, Andrea Bottari, Laura Fortuna, Jacopo Martellucci, Atea Cicako, Claudio Miglietta, Mario Morino, Daniele Delogu, Andrea Picchetto, Marco Assenza, Giancarlo D'Ambrosio, Giulio Argenio, Mariano Fortunato Armellino, Giovanna Ioia, Savino Occhionorelli, Dario Andreotti, Lacavalla Domenico, Davide Luppi, Massimiliano Casadei, Luca Di Donato, Farshad Manoochehri, Tiziana Rita Lucia Marchese, William Sergi, Roberto Manca, Raimondo Murgia, Enrico Piras, Lorenzo Conti, Simone Gianazza, Andrea Rizzi, Edoardo Segalini, Marco Monti, Elena Iiritano, Nicolò Maria Mariani, Enrico De Nicola, Giovanna Scifo, Giusto Pignata, Jacopo Andreuccetti, Francesco Fleres, Guglielmo Clarizia, Alessandro Spolini, Alan Biloslavo, Paola Germani, Manuela Mastronardi, Selene Bogoni, Silvia Palmisano, Nicolo’ De Manzini, Marco Vito Marino, Gennaro Martines, Giuseppe Trigiante, Elpiniki Lagouvardou, Gabriele Anania, Cristina Bombardini, Dario Oppici, Tiziana Pilia, Valentina Murzi, Emanuela Gessa, Umberto Bracale, Maria Michela Di Nuzzo, Roberto Peltrini, Francesco Salvetti, Jacopo Viganò, Gabriele Sganga, Valentina Bianchi, Pietro Fransvea, Tommaso Fontana, Giuliano Sarro, Vincenza Paola Dinuzzi, Luca Scaravilli, Mario Virgilio Papa, Elio Jovine, Giulia Ciabatti, Laura Mastrangelo, Matteo Rottoli, Claudio Ricci, Iris Shari Russo, Alberto Aiolfi, Davide Bona, Francesca Lombardo, Pasquale Cianci, Mariagrazia Sederino, Roberto Bini, Osvaldo Chiara, Stefano Cioffi, Stefano Cantafio, Guido Coretti, Edelweiss Licitra, Grazia Savino, Sergio Grimaldi, Raffaele Porfidia, Elisabetta Moggia, Mauro Garino, Chiara Marafante, Antonio Pesce, Nicolò Fabbri, Carlo Vittorio Feo, Ester Marra, Marina Troian, Davide Drigo, Carlo Nagliati, Andrea Muratore, Riccardo Danna, Alessandra Murgese, Michele Crespi, Claudio Guerci, Alice Frontali, Luca Ferrari, Claudio Guerci, Francesco Favi, Erika Picariello, Alessia Rampini, Fabrizio D'Acapito, Giorgio Ercolani, Leonardo Solaini, Francesco Palmieri, Matteo Calì, Francesco Ferrara, Irnerio Angelo Muttillo, Edoardo Maria Muttillo, Biagio Picardi, Raffaele Galleano, Ali Badran, Omar Ghazouani, Maurizio Cervellera, Gaetano Campanella, Gennaro Papa, Annamaria Di Bella, Gennaro Perrone, Gabriele Luciano Petracca, Concetta Prioriello, Mario Giuffrida, Federico Cozzani, Matteo Rossini, Marco Inama, Giovanni Butturini, Gianluigi Moretto, Luca Morelli, Giulio Di Candio, Simone Guadagni, Enrico Cicuttin, Camilla Cremonini, Dario Tartaglia, Valerio Genovese, Nicola Cillara, Alessandro Cannavera, Antonello Deserra, Arcangelo Picciariello, Vincenzo Papagni, Leonardo Vincenti, Giulia Bagaglini, Giuseppe Sica, Pierfrancesco Lapolla, Gioia Brachini, Dario Bono, Antonella Nicotera, Marcello Zago, Fabrizio Sammartano, Laura Benuzzi, Marco Stella, Stefano Rossi, Alessandra Cerioli, Caterina Puccioni, Stefano Olmi, Carolina Rubicondo, Matteo Uccelli, Andrea Balla, Anna Guida, Pasquale Lepiane, Diego Sasia, Giorgio Giraudo, Sara Salomone, Elena Belloni, Alessandra Cossa, Francesco Lancellotti, Roberto Caronna, Piero Chirletti, Paolina Saullo, Raffaele Troiano, Felice Mucilli, Mirko Barone, Massimo Ippoliti, Michele Grande, Bruno Sensi, Leandro Siragusa, Monica Ortenzi, Andrea Santini, Isidoro Di Carlo, Massimiliano Veroux, Rossella Gioco, Gastone Veroux, Giuseppe Currò, Michele Ammendola, Iman Komaei, Giuseppe Navarra, Valeria Tonini, Lodovico Sartarelli, Samuele Vaccarim Marco Ceresoli, Stefano Perrone, Linda Roccamatisi, Paolo Millo, Riccardo Brachet Contul, Elisa Ponte, Matteo Zuin, Giuseppe Portale, Alice Sabrina Tonello, Geri Fratini, Matteo Bianchini, Bruno Perotti, Emanuele Doria, Elia Giuseppe Lunghi, Diego Visconti, Khayry Al-Shami, Sajeda Awadi, Mohammad Musallam Khalil Buwaitel, Mo'taz Fawzat Naief Naffa', Ahmad Samhouri, Hatem Sawalha, Mohd Firdaus Che Ani, Ida Nadiah Ahmed Fathil, Jih Huei, Ikhwan Sani Mohamad, Andee Dzulkarnaen Zakaria, Mohammad Zawawi Ya'acob, Jose-Luis Beristain-Hernandez, Alejandro Garcia-Meza, Rafael Sepulveda-Rdriguez, Edgard Efren Lozada Hernández, Camilo Levi Acuña Pinzón, Jefferson Nieves Condoy, Francisco C. Becerra García, Mohammad Sadik, Bushra Kadir, Jalpa Devi, Nandlal Seerani, Zainab, Mohammad Sohail- Asghar, Ameer Afzal, Ali Akbar, Helmut Segovia Lohse, Herald Segovia Lohse, Zamiara Solange Leon Cabrera, Gaby Susana Yamamoto Seto, José Ríos Chiuyari, Jorge Ordemar, Martha Rodríguez, Abigail Cheska C. Orantia-Carlos, Margie Antionette Quitoy, Andrzej Kwiatkowski, Maciej Mawlichanów, Mónica Rocha, Carlos Soares, Alexandru Rares Stoian, Andreea Diana Draghici, Valentin Titus Grigorean, Raluca Bievel Radulescu, Narcis Octavian Zarnescu, Radu Virgil Costea, Eugenia Claudia Zarnescu, Mikhail Kurtenkov, George Gendrikson, Volovich Alla-Angelina, Tsurbanova Arina, Ayrat Kaldarov, Ayrat Kaldarov, Mahir Gachabayov, Abakar Abdullaev, Milica Milentijevic, Milovan Karamarkovic, Arpád Panyko, Jozef Radonak, Marek Soltes, Laura Álvarez Morán, Haydée Calvo García, Pilar Suárez Vega, Sergio Estevez, Fabio Ausania, Jordi Farguell, Carolina González-Abós, Santiago Sánchez-Cabús, Belén Martín, Víctor Molina, Luis Oms, Lucas Ilzarbe, Eva Pont Feijóo, Elena Sofia Perra, Noel Rojas-Bonet, Rafael Penalba-Palmí, Susana Pérez-Bru, Jaume Tur-Martínez, Andrea Álvarez-Torrado, Marta Domingo-Gonzalez, Javier Tejedor-Tejada, Marcello Di Martino, Yaiza García del Alamo, Fernando Mendoza-Moreno, Francisca García-Moreno-Nisa, Belén Matías-García, Manuel Durán, Rafael Calleja-Lozano, José Manuel Perez de Villar, Luis Sánchez-Guillén, Iban Caravaca, Daniel Triguero-Cánovas, Antonio Carlos Maya Aparicio, Blas Durán Meléndez, Andrea Masiá Palacios, Aitor Landaluce-Olavarria, Mario De Francisco, Begoña Estraviz-Mateos, Felipe Alconchel, Tatiana Nicolás-López, Pablo Ramírez, Virginia Duran Muñoz-Cruzado, Felipe Pareja Ciuró, Eduardo Perea del Pozo, Sergio Olivares Pizarro, Vicente Herrera Cabrera, Jose Muros Bayo, Hytham K. S. Hamid, Raffaello Roesel, Alessandra Cristaudi, Kinan Abbas, Iyad Ali, Ahmed Tlili, Hüseyin Bayhan, Mehmet Akif Türkoğlu, Mustafa Yener Uzunoglu, Ibrahim Fethi Azamat, Nail Omarov, Derya Salim Uymaz, Fatih Altintoprak, Emrah Akin, Necattin First, Koray Das, Nazmi Ozer, Ahmet Seker, Yasin Kara, Mehmet Abdussamet Bozkurt, Ali Kocataş, Semra Demirli Atici, Murat Akalin, Bulent Calik, Elif Colak, Yuksel Altinel, Serhat Meric, Yunus Emre Aktimur, Victoria Hudson, Jean-Luc Duval, Mansoor Khan, Ahmed Saad, Mandeep Kaur, Alison Bradley, Katherine Fox, Ivan Tomasi, Daniel Beasley, Alekhya Kotta Prasanti, Pinky Kotecha, Husam Ebied, Michaela Paul, Hemant Sheth, Ioannis Gerogiannis, Mohannad Gaber, Zara Sheikh, Shatadru Seth, Maria Kunitsyna, Cosimo Alex Leo, Vittoria Bellato, Noman – Zafar, Amr Elserafy, Giles Bond-smith, Giovanni Tebala, Pawan Mathur, Izza Abid, Nnaemeka Chidumije, Pardip Sandhar, Syed Osama Zohaib Ullah, Tamara Lezama, Muhammad Hassan Anwaar, Conor Magee, Salma Ahmed, Brooke Davies, Jeyakumar Apollos, Kieran McCormack, Hasham Choudhary, Triantafyllos Doulias, Tamsin Morrison, Anna Palepa, Fernando Bonilla Cal, Lianet Sánchez, Fabiana Domínguez, Ibrahim Al-Raimi, Haneen Alshargabi, Abdullah Meead, Serge Chooklin, Serhii Chuklin, Andriy Bilyak.
