# Supplementary material for: Mild acute biliary pancreatitis: still a surgical disease. A post-hoc analysis of the MANCTRA-1 international study
Source: Eur J Trauma Emerg Surg. 2025 Jan 17;51(1):24. doi: 10.1007/s00068-024-02748-9 (PMC11742350; doi:10.1007/s00068-024-02748-9)
Supplement: Supplementary file 2 [file 68_2024_2748_MOESM2_ESM.docx]

| ***First Name and Middle Initial(s)** | ***Last Name** | | Academic Degrees | Institution | | | Location (city, state/province, country) | | Role or Contribution, | |  |
| --- | --- | --- | --- | --- | --- | --- | --- | --- | --- | --- | --- |
| Chiara | Gerardi | | Ph.D. Pharm.D. | Istituto di Ricerche Farmacologiche "Mario Negri" IRCCS | | | Milan, Italy | | Methodology advisor | |  |
| Francesco | Virdis | | MD. | Trauma and Acute Care Surgery Unit, Niguarda Ca Granda Hospital | | | Milan, Italy | | Clinical advisor | |  |
| Daniela | Pacella | | Ph.D. | University of Naples Federico II, Department of Public Health | | | Naples, Italy | | Statistical advisor | |  |
| Kumar | Jayant | | MD. | Department of Surgery, Transplant Division, University of Chicago | | | Chicago, IL, USA | | Clinical advisor | |  |
| Ferdinando | Agresta | | MD. | Department of Surgery, Vittorio Veneto Civil Hospital | | | Vittorio Veneto, Italy | | Clinical advisor | |  |
| Cristiana | Riboni | | MD. | Department of Surgery, EOC Regional Hospital | | | Lugano, Switzerland | | Clinical advisor | |  |
| Yoram | Kluger | | MD. | Division of General Surgery, Rambam Health Care Campus | | | Haifa, Israel | | Clinical advisor | |  |
| Martin | de Santibañes | | MD. | Hospital Italiano de Buenos Aires | | | Buenos Aires, Argentina | | Local investigator | |  |
| Juliana | Di Menno Stavron | MD. Hospital Italiano de Buenos Aires | | |  | Buenos Aires, Argentina | | Local investigator | |  |  |
| Oscar | Mazza | | MD. | Hospital Italiano de Buenos Aires | | | Buenos Aires, Argentina | | Local investigator | |  |
| José I | Valenzuela | | MD. | Hospital Velez Sarsfield | | | Buenos Aires, Argentina | | Local investigator | |  |
| Diana AP | Pachajoa | | MD. | Clinica Universitaria Reina Fabiola | | | Cordoba, Argentina | | Local investigator | |  |
| Fernando A | Alvarez | | MD. | Clinica Universitaria Reina Fabiola | | | Cordoba, Argentina | | Local investigator | |  |
| Julian E | Liaño | | MD. | Clinica Universitaria Reina Fabiola | | | Cordoba, Argentina | | Local investigator | |  |
| Justin | Gundara | | MD. | Logan Hospital, Metro South Health | | | Meadowbrook, Australia | | Local investigator | |  |
| Joan | Tefay | | MD. | Redland Hospital | | | Redland City, Australia | | Local investigator | |  |
| Kamel | Alawadhi | | MD. | Salmaniya Medical Complex | | | Manama, Bahrain | | Local investigator | |  |
| Abdulrahman | Alshaikh | | MD. | Salmaniya Medical Complex | | | Manama, Bahrain | | Local investigator | |  |
| Layla | Hasan | | MD. | Salmaniya Medical Complex | | | Manama, Bahrain | | Local investigator | |  |
| Carlos A | Gomes | | MD. | Hospital Universitario Terezinha de Jesus. Faculdade de Medicina Suprema | | | Juiz de Fora, Brasil | | Local investigator | |  |
| Felipe C | Gomes | | MD. | Hospital Universitario Terezinha de Jesus. Faculdade de Medicina Suprema | | | Juiz de Fora, Brasil | | Local investigator | |  |
| Gustavo P | Fraga | | MD. | University of Campinas | | | Campinas, Brasil | | Local investigator | |  |
| Thiago RA | Calderan | | MD. | University of Campinas | | | Campinas, Brasil | | Local investigator | |  |
| Elcio S | Hirano | | MD. | University of Campinas | | | Campinas, Brasil | | Local investigator | |  |
| Dragomir | Dardanov | | MD. | Alexandrovska University Hospital | | | Sofia, Bulgaria | | Local investigator | |  |
| Alexander | Julianov | | MD. | Trakia Hospital | | | Stara Zagora, Bulgaria | | Local investigator | |  |
| Azize | Saroglu | | MD. | Trakia Hospital | | | Stara Zagora, Bulgaria | | Local investigator | |  |
| Boyko | Atanasov | | MD. | UMHAT Eurohospital, Medical University Plovdiv | | | Plovdiv, Bulgaria | | Local investigator | |  |
| Nikolay | Belev | | MD. | UMHAT Eurohospital, Medical University Plovdiv | | | Plovdiv, Bulgaria | | Local investigator | |  |
| Nikola | Kovachev | | MD. | UMHAT Eurohospital, Medical University Plovdiv | | | Plovdiv, Bulgaria | | Local investigator | |  |
| Rashid | Lui | | MD. | The Chinese University of Hong Kong | | | Hong Kong, China | | Local investigator | |  |
| Shannon M | Chan | | MD. | The Chinese University of Hong Kong | | | Hong Kong, China | | Local investigator | |  |
| Hon-Ting | Lok | | MD. | The Chinese University of Hong Kong | | | Hong Kong, China | | Local investigator | |  |
| Diego | Salcedo | | MD. | Hospital Simón Bolívar | | | Bogotà, Colombia | | Local investigator | |  |
| Diana | Robayo | | MD. | Hospital Simón Bolívar | | | Bogotà, Colombia | | Local investigator | |  |
| Maria A | Triviño | | MD. | Hospital Simón Bolívar | | | Bogotà, Colombia | | Local investigator | |  |
| Jan | Manak | | MD. | Faculty Hospital Hradec Kralove | | | Hradec Kralove, Czech Republic | | Local investigator | |  |
| Saaz | Sahani | | MD. | Faculty Hospital Hradec Kralove | | | Hradec Kralove, Czech Republic | | Local investigator | |  |
| Jorann | de Araujo | | MD. | Faculty Hospital Hradec Kralove | | | Hradec Kralove, Czech Republic | | Local investigator | |  |
| Ananya | Sethi | | MD. | Faculty Hospital Hradec Kralove | | | Hradec Kralove, Czech Republic | | Local investigator | |  |
| Ahmed | Awad | | MD. | Ain-Shams University Hospitals | | | Heliopolis, Egypt | | Local investigator | |  |
| Merihan | Elbadawy | | MD. | Ain-Shams University Hospitals | | | Heliopolis, Egypt | | Local investigator | |  |
| Ahmed | Farid | | MD. | Alexandria University | | | Alexandria, Egypt | | Local investigator | |  |
| Asmaa | Hanafy | | MD. | Alexandria University | | | Alexandria, Egypt | | Local investigator | |  |
| Ahmed | Nafea | | MD. | Alexandria University | | | Alexandria, Egypt | | Local investigator | |  |
| Sherief | Ghozy | | MD. | El Sheikh Zayed Specialized Hospital | | | Giza, Egypt | | Local investigator | |  |
| Alzhraa | Salah–Abbas | | MD. | El Sheikh Zayed Specialized Hospital | | | Giza, Egypt | | Local investigator | |  |
| Wafaa | Abdelsalam | | MD. | KafrElsheikh University Faculty of Medicine | | | Kafr-El-sheikh, Egypt | | Local investigator | |  |
| Sameh | Emile | | MD. | Mansoura University Hospital | | | El Mansoura, Egypt | | Local investigator | |  |
| Ahmed | Elfallal | | MD. | Mansoura University Hospital | | | El Mansoura, Egypt | | Local investigator | |  |
| Hossam | Elfeki | | MD. | Mansoura University Hospital | | | El Mansoura, Egypt | | Local investigator | |  |
| Hosam | Elghadban | | MD. | Mansoura University Hospital | | | El Mansoura, Egypt | | Local investigator | |  |
| Ashraf | Shoma | | MD. | Mansoura University Hospital | | | El Mansoura, Egypt | | Local investigator | |  |
| Mohamed | Shetiwy | | MD. | Mansoura University Hospital | | | El Mansoura, Egypt | | Local investigator | |  |
| Mohamed | Elbahnasawy | | MD. | Tanta University | | | El Gaish, Egypt | | Local investigator | |  |
| Salem | Mohamed | | MD. | Zagazig University | | | Zagazig, Egypt | | Local investigator | |  |
| Emad F | Hamed | | MD. | Zagazig University | | | Zagazig, Egypt | | Local investigator | |  |
| Usama A | Khalil | | MD. | Zagazig University | | | Zagazig, Egypt | | Local investigator | |  |
| Elie | Chouillard | | MD. | Centre Hospitalier Intercommunal de Poissy/Saint Germain en Laye | | | Saint Germain en Laye, France | | Local investigator | |  |
| Andrew | Gumbs | | MD. | Centre Hospitalier Intercommunal de Poissy/Saint Germain en Laye | | | Saint Germain en Laye, France | | Local investigator | |  |
| Andréa | Police | | MD. | Hôpital Simone Veil | | | Eaubonne, France | | Local investigator | |  |
| Andrea | Mabilia | | MD. | Hôpital Simone Veil | | | Eaubonne, France | | Local investigator | |  |
| Zaza | Demetrashvili | | MD. | Kipshidze Central University Hospital | | | Tbilisi, Georgia | | Local investigator | |  |
| Kakhi | Khutsishvili | | MD. | Kipshidze Central University Hospital | | | Tbilisi, Georgia | | Local investigator | |  |
| Anano | Tvaladze | | MD. | Kipshidze Central University Hospital | | | Tbilisi, Georgia | | Local investigator | |  |
| Orestis | Ioannidis | | MD. | Aristotle University of Thessaloniki | | | Thessaloniki, Greece | | Local investigator | |  |
| Elissavet | Anestiadou | | MD. | Aristotle University of Thessaloniki | | | Thessaloniki, Greece | | Local investigator | |  |
| Lydia | Loutzidou | | MD. | Aristotle University of Thessaloniki | | | Thessaloniki, Greece | | Local investigator | |  |
| Argyrios | Ioannidis | | MD. | Athens Medical Center | | | Athens, Greece | | Local investigator | |  |
| Konstantinis | Konstantinidis | | MD. | Athens Medical Center | | | Athens, Greece | | Local investigator | |  |
| Sofia | Konstantinidou | | MD. | Athens Medical Center | | | Athens, Greece | | Local investigator | |  |
| Dimitrios | Manatakis | | MD. | Athens Naval and Veterans Hospital | | | Athens, Greece | | Local investigator | |  |
| Vasileios | Acheimastos | | MD. | Athens Naval and Veterans Hospital | | | Athens, Greece | | Local investigator | |  |
| Nikolaos | Tasis | | MD. | Athens Naval and Veterans Hospital | | | Athens, Greece | | Local investigator | |  |
| Nikolaos | Michalopoulos | | MD. | Attikon General University Hospital of Athens | | | Athens, Greece | | Local investigator | |  |
| Panagiotis | Kokoropoulos | | MD. | Attikon General University Hospital of Athens | | | Athens, Greece | | Local investigator | |  |
| Maria | Papadoliopoulou | | MD. | Attikon General University Hospital of Athens | | | Athens, Greece | | Local investigator | |  |
| Maria | Sotiropoulou | | MD. | Evangelismos General Hospital | | | Athens, Greece | | Local investigator | |  |
| Stylianos | Kapiris | | MD. | Evangelismos General Hospital | | | Athens, Greece | | Local investigator | |  |
| Panagiotis | Metaxas | | MD. | Evangelismos General Hospital | | | Athens, Greece | | Local investigator | |  |
| Ioannis | Tsouknidas | | MD. | General Hospital of Chania "Agios Georgios" | | | Crete, Greece | | Local investigator | |  |
| Despoina | Kefili | | MD. | General Hospital of Chania "Agios Georgios" | | | Crete, Greece | | Local investigator | |  |
| George | Petrakis | | MD. | General Hospital of Chania "Agios Georgios" | | | Crete, Greece | | Local investigator | |  |
| Eirini | Synekidou | | MD. | General Hospital of Kozani | | | Kozani, Greece | | Local investigator | |  |
| Konstantinos | Dakis | | MD. | General Hospital of Kozani | | | Kozani, Greece | | Local investigator | |  |
| Eirini | Alexandridou | | MD. | General Hospital of Kozani | | | Kozani, Greece | | Local investigator | |  |
| Aristeidis | Papadopoulos | | MD. | General Hospital of Nikaia | | | Nikaia, Greece | | Local investigator | |  |
| Christos | Chouliaras | | MD. | General Hospital of Nikaia | | | Nikaia, Greece | | Local investigator | |  |
| Odysseas | Mouzakis | | MD. | General Hospital of Nikaia | | | Nikaia, Greece | | Local investigator | |  |
| Francesk | Mulita | | MD. | General University Hospital of Patras | | | Patras, Greece | | Local investigator | |  |
| Ioannis | Maroulis | | MD. | General University Hospital of Patras | | | Patras, Greece | | Local investigator | |  |
| Michail | Vailas | | MD. | General University Hospital of Patras | | | Patras, Greece | | Local investigator | |  |
| Tania | Triantafyllou | | MD. | Hippocration General Hospital of Athens, University of Athens | | | Athens, Greece | | Local investigator | |  |
| Dimitrios | Theodorou | | MD. | Hippocration General Hospital of Athens, University of Athens | | | Athens, Greece | | Local investigator | |  |
| Eftychios | Lostoridis | | MD. | Kavala General Hospital | | | Kavala, Greece | | Local investigator | |  |
| Eleni A | Nagorni | | MD. | Kavala General Hospital | | | Kavala, Greece | | Local investigator | |  |
| Paraskevi | Tourountzi | | MD. | Kavala General Hospital | | | Kavala, Greece | | Local investigator | |  |
| Efstratia | Baili | | MD. | IASO Hospital, Athens | | | Athens, Greece | | Local investigator | |  |
| Alexandros | Charalabopoulos | | MD. | IASO Hospital, Athens | | | Athens, Greece | | Local investigator | |  |
| Theodore | Liakakos | | MD. | IASO Hospital, Athens | | | Athens, Greece | | Local investigator | |  |
| Dimitrios | Schizas | | MD. | 1st Department of Surgery, Laiko General Hospital | | | Athens, Greece | | Local investigator | |  |
| Alexandros | Kozadinos | | MD. | 1st Department of Surgery, Laiko General Hospital | | | Athens, Greece | | Local investigator | |  |
| Athanasios | Syllaios | | MD. | 1st Department of Surgery, Laiko General Hospital | | | Athens, Greece | | Local investigator | |  |
| Nikolaos | Machairas | | MD. | 2nd Department of Propaedeutic Surgery, Laiko General Hospital | | | Athens, Greece | | Local investigator | |  |
| Stylianos | Kykalos | | MD. | 2nd Department of Propaedeutic Surgery, Laiko General Hospital | | | Athens, Greece | | Local investigator | |  |
| Paraskevas | Stamopoulos | | MD. | 2nd Department of Propaedeutic Surgery, Laiko General Hospital | | | Athens, Greece | | Local investigator | |  |
| Spiros | Delis | | MD. | St Olga Hospital, University of Athens | | | Athens, Greece | | Local investigator | |  |
| Christos | Farazi-Chongouki | | MD. | Thriasio General Hospital of Elefsina | | | Athens, Greece | | Local investigator | |  |
| Evangelos | Kalaitzakis | | MD. | Department of Gastroenterolgy, University Hospital of Heraklion | | | Heraklion, Greece | | Local investigator | |  |
| Miltiadis | Giannarakis | | MD. | Department of Gastroenterolgy, University Hospital of Heraklion | | | Heraklion, Greece | | Local investigator | |  |
| Konstantinos | Lasithiotakis | | MD. | Department of General Surgery, University Hospital of Heraklion | | | Heraklion, Greece | | Local investigator | |  |
| Giorgia | Petra | | MD. | Department of General Surgery, University Hospital of Heraklion | | | Heraklion, Greece | | Local investigator | |  |
| Evangelos | Kalaitzakis | | MD. | Department of General Surgery, University Hospital of Heraklion | | | Heraklion, Greece | | Local investigator | |  |
| Nikolaos | Koronakis | | MD. | ICU Nicosia General Hospital | | | Nicosia, Ciprus | | Local investigator | |  |
| Sergio | Ralon | | MD. | Universidad de San Carlos de Guatemala, Hospital General San Juan de Dios | | | Ciudad de Guatemala, Guatemala | | Local investigator | |  |
| Amit | Gupta | | MD. | AIIMS Rishikesh | | | Rishikesh, India | | Local investigator | |  |
| Noushif | Medappil | | MD. | Aster Malabar Institute of Medical Sciences, Aster MIMS | | | Kozhicode, India | | Local investigator | |  |
| Vijayanand | Muthukrishnan | | MD. | Aster Malabar Institute of Medical Sciences, Aster MIMS | | | Kozhicode, India | | Local investigator | |  |
| Jubin | Kamar | | MD. | Aster Malabar Institute of Medical Sciences, Aster MIMS | | | Kozhicode, India | | Local investigator | |  |
| Lovenish | Bains | | MD. | Maulana Azad Medical College, New Delhi | | | New Delhi, India | | Local investigator | |  |
| Pawan | Lal | | MD. | Maulana Azad Medical College, New Delhi | | | New Delhi, India | | Local investigator | |  |
| Rajendra | Agarwal | | MD. | Sudha Hospital and Medical Research Centre | | | Kota, India | | Local investigator | |  |
| Matteo | Magnoli | | MD. | Acqui Terme Hospital | | | Acqui Terme, Italy | | Local investigator | |  |
| Paolo | Aonzo | | MD. | Acqui Terme Hospital | | | Acqui Terme, Italy | | Local investigator | |  |
| Alberto | Serventi | | MD. | Acqui Terme Hospital | | | Acqui Terme, Italy | | Local investigator | |  |
| Antonio | Giuliani | | MD. | AOR San Carlo Potenza | | | Potenza, Italy | | Local investigator | |  |
| Pierpaolo | Di Lascio | | MD. | AOR San Carlo Potenza | | | Potenza, Italy | | Local investigator | |  |
| Margherita | Pinto | | MD. | AOR San Carlo Potenza | | | Potenza, Italy | | Local investigator | |  |
| Carlo | Bergamini | | MD. | AOU Careggi | | | Firenze, Italy | | Local investigator | |  |
| Andrea | Bottari | | MD. | AOU Careggi | | | Firenze, Italy | | Local investigator | |  |
| Laura | Fortuna | | MD. | AOU Careggi | | | Firenze, Italy | | Local investigator | |  |
| Jacopo | Martellucci | | MD. | AOU Careggi | | | Firenze, Italy | | Local investigator | |  |
| Atea | Cicako | | MD. | AOU Città della Salute e della Scienza di Torino - San Giovanni Battista – Molinette | | | Torino, Italy | | Local investigator | |  |
| Claudio | Miglietta | | MD. | AOU Città della Salute e della Scienza di Torino - San Giovanni Battista – Molinette | | | Torino, Italy | | Local investigator | |  |
| Mario | Morino | | MD. | AOU Città della Salute e della Scienza di Torino - San Giovanni Battista – Molinette | | | Torino, Italy | | Local investigator | |  |
| Daniele | Delogu | | MD. | AOU di Sassari, Cliniche Universitarie di San Pietro | | | Sassari, Italy | | Local investigator | |  |
| Andrea | Picchetto | | MD. | AOU Policlinico Umberto I | | | Roma, Italy | | Local investigator | |  |
| Marco | Assenza | | MD. | AOU Policlinico Umberto I | | | Roma, Italy | | Local investigator | |  |
| Giancarlo | D'Ambrosio | | MD. | AOU Policlinico Umberto I | | | Roma, Italy | | Local investigator | |  |
| Giulio | Argenio | | MD. | AOU San Giovanni di Dio e Ruggi d'Aragona | | | Salerno, Italy | | Local investigator | |  |
| Giovanna | Ioia | | MD. | AOU San Giovanni di Dio e Ruggi d'Aragona | | | Salerno, Italy | | Local investigator | |  |
| Mariano F | Armellino | | MD. | AOU San Giovanni di Dio e Ruggi d'Aragona | | | Salerno, Italy | | Local investigator | |  |
| Savino | Occhionorelli | | MD. | Arcispedale Sant'Anna, University of Ferrara | | | Ferrara, Italy | | Local investigator | |  |
| Domenico | Lacavalla | | MD. | Arcispedale Sant'Anna, University of Ferrara | | | Ferrara, Italy | | Local investigator | |  |
| Dario | Andreotti | | MD. | Arcispedale Sant'Anna, University of Ferrara | | | Ferrara, Italy | | Local investigator | |  |
| Davide | Luppi | | MD. | Arcispedale Santa Maria Nuova - AUSL-IRCCS Reggio Emilia | | | Reggio Emilia, Italy | | Local investigator | |  |
| Massimiliano | Casadei | | MD. | Arcispedale Santa Maria Nuova - AUSL-IRCCS Reggio Emilia | | | Reggio Emilia, Italy | | Local investigator | |  |
| Luca | Di Donato | | MD. | Arcispedale Santa Maria Nuova - AUSL-IRCCS Reggio Emilia | | | Reggio Emilia, Italy | | Local investigator | |  |
| Farshad | Manoochehri | | MD. | Asl Lecce P.O. Vito Fazzi | | | Lecce, Italy | | Local investigator | |  |
| Tiziana RL | Marchese | | MD. | Asl Lecce P.O. Vito Fazzi | | | Lecce, Italy | | Local investigator | |  |
| William | Sergi | | MD. | Asl Lecce P.O. Vito Fazzi | | | Lecce, Italy | | Local investigator | |  |
| Roberto | Manca | | MD. | ASSL Cagliari ATS Sardegna, UOC Gastroenterologia ed Endoscopia Digestiva | | | Cagliari, Italy | | Local investigator | |  |
| Raimondo | Murgia | | MD. | ASSL Cagliari ATS Sardegna, UOC Gastroenterologia ed Endoscopia Digestiva | | | Cagliari, Italy | | Local investigator | |  |
| Enrico | Piras | | MD. | ASSL Cagliari ATS Sardegna, UOC Gastroenterologia ed Endoscopia Digestiva | | | Cagliari, Italy | | Local investigator | |  |
| Lorenzo | Conti | | MD. | ASST dei Sette Laghi - Ospedale Galmarini | | | Varese, Italy | | Local investigator | |  |
| Simone | Gianazza | | MD. | ASST dei Sette Laghi - Ospedale Galmarini | | | Varese, Italy | | Local investigator | |  |
| Andrea | Rizzi | | MD. | ASST dei Sette Laghi - Ospedale Galmarini | | | Varese, Italy | | Local investigator | |  |
| Edoardo | Segalini | | MD. | ASST Ospedale Maggiore Crema | | | Crema, Italy | | Local investigator | |  |
| Marco | Monti | | MD. | ASST Ospedale Maggiore Crema | | | Crema, Italy | | Local investigator | |  |
| Elena | Liritano | | MD. | ASST Ospedale Maggiore Crema | | | Crema, Italy | | Local investigator | |  |
| Nicolò M | Mariani | | MD. | ASST Santi Paolo e Carlo | | | Milan, Italy | | Local investigator | |  |
| Enrico | De Nicola | | MD. | ASST Santi Paolo e Carlo | | | Milan, Italy | | Local investigator | |  |
| Giovanna | Scifo | | MD. | ASST Santi Paolo e Carlo | | | Milan, Italy | | Local investigator | |  |
| Giusto | Pignata | | MD. | ASST Spedali Civili di Brescia | | | Brescia, Italy | | Local investigator | |  |
| Jacopo | Andreuccetti | | MD. | ASST Spedali Civili di Brescia | | | Brescia, Italy | | Local investigator | |  |
| Francesco | Fleres | | MD. | ASST Valtellina e Alto Lario, Sondrio Hospital | | | Sondrio, Italy | | Local investigator | |  |
| Guglielmo | Clarizia | | MD. | ASST Valtellina e Alto Lario, Sondrio Hospital | | | Sondrio, Italy | | Local investigator | |  |
| Alessandro | Spolini | | MD. | ASST Valtellina e Alto Lario, Sondrio Hospital | | | Sondrio, Italy | | Local investigator | |  |
| Alan | Biloslavo | | MD. | ASUGI Clinica Chirurgica Trieste | | | Trieste, Italy | | Local investigator | |  |
| Paola | Germani | | MD. | ASUGI Clinica Chirurgica Trieste | | | Trieste, Italy | | Local investigator | |  |
| Manuela | Mastronardi | | MD. | ASUGI Clinica Chirurgica Trieste | | | Trieste, Italy | | Local investigator | |  |
| Selene | Bogoni | | MD. | ASUGI Clinica Chirurgica Trieste | | | Trieste, Italy | | Local investigator | |  |
| Silvia | Palmisano | | MD. | ASUGI Clinica Chirurgica Trieste | | | Trieste, Italy | | Local investigator | |  |
| Nicolò | De Manzini | | MD. | ASUGI Clinica Chirurgica Trieste | | | Trieste, Italy | | Local investigator | |  |
| Marco V | Marino | | MD. | Azienda Ospedaliera Ospedali Riuniti Villa Sofia Cervello Palermo | | | Palermo, Italy | | Local investigator | |  |
| Gaetano | Poillucci | | MD. | Policlinico Universitario Umberto I Roma, Emergency Surgery Unit | | | Roma, Italy | | Local investigator | |  |
| Gennaro | Martines | | MD. | Azienda Ospedaliera Universitaria Policlinico Bari | | | Bari, Italy | | Local investigator | |  |
| Giuseppe | Trigiante | | MD. | Azienda Ospedaliera Universitaria Policlinico Bari | | | Bari, Italy | | Local investigator | |  |
| Elpiniki | Lagouvardou | | MD. | Azienda Ospedaliera Universitaria Policlinico Bari | | | Bari, Italy | | Local investigator | |  |
| Gabriele | Anania | | MD. | Azienda Ospedaliero-Universitaria di Ferrara | | | Ferrara, Italy | | Local investigator | |  |
| Cristina | Bombardini | | MD. | Azienda Ospedaliero-Universitaria di Ferrara | | | Ferrara, Italy | | Local investigator | |  |
| Dario | Oppici | | MD. | Azienda Ospedaliero-Universitaria di Ferrara | | | Ferrara, Italy | | Local investigator | |  |
| Tiziana | Pilia | | MD. | Cagliari University Hospital | | | Cagliari, Italy | | Local investigator | |  |
| Valentina | Murzi | | MD. | Cagliari University Hospital | | | Cagliari, Italy | | Local investigator | |  |
| Emanuela | Gessa | | MD. | Cagliari University Hospital | | | Cagliari, Italy | | Local investigator | |  |
| Eleonora | Locci | | MD. | Cagliari University Hospital | | | Cagliari, Italy | | Local investigator | |  |
| Umberto | Bracale | | MD. | Federico II University Hospital | | | Napoli, Italy | | Local investigator | |  |
| Maria M | Di Nuzzo | | MD. | Federico II University Hospital | | | Napoli, Italy | | Local investigator | |  |
| Roberto | Peltrini | | MD. | Federico II University Hospital | | | Napoli, Italy | | Local investigator | |  |
| Luca | Ansaloni | | MD. | Fondazione IRCCS Policlinico San Matteo | | | Pavia, Italy | | Local investigator | |  |
| Francesco | Salvetti | | MD. | Fondazione IRCCS Policlinico San Matteo | | | Pavia, Italy | | Local investigator | |  |
| Jacopo | Viganò | | MD. | Fondazione IRCCS Policlinico San Matteo | | | Pavia, Italy | | Local investigator | |  |
| Gabriele | Sganga | | MD. | Fondazione Policlinico Universitario A. Gemelli IRCC, Università Cattolica de Sacro Cuore | | | Roma, Italy | | Local investigator | |  |
| Valentina | Bianchi | | MD. | Fondazione Policlinico Universitario A. Gemelli IRCC, Università Cattolica de Sacro Cuore | | | Roma, Italy | | Local investigator | |  |
| Pietro | Fransvea | | MD. | Fondazione Policlinico Universitario A. Gemelli IRCC, Università Cattolica de Sacro Cuore | | | Roma, Italy | | Local investigator | |  |
| Tommaso | Fontana | | MD. | “G.F. Ingrassia” Hospital | | | Catania, Italy | | Local investigator | |  |
| Giuliano | Sarro | | MD. | G.Fornaroli Hospital, ASST Ovest Milanese, Magenta | | | Magenta, Italy | | Local investigator | |  |
| Vincenza P | Dinuzzi | | MD. | G.Fornaroli Hospital, ASST Ovest Milanese, Magenta | | | Magenta, Italy | | Local investigator | |  |
| Luca | Scaravilli | | MD. | G.Fornaroli Hospital, ASST Ovest Milanese, Magenta | | | Magenta, Italy | | Local investigator | |  |
| Mario V | Papa | | MD. | Hospital of Caserta | | | Caserta, Italy | | Local investigator | |  |
| Elio | Jovine | | MD. | IRCCS Azienda Ospedaliera Universitaria di Bologna c/o Ospedale Maggiore | | | Bologna, Italy | | Local investigator | |  |
| Giulia | Ciabatti | | MD. | IRCCS Azienda Ospedaliera Universitaria di Bologna c/o Ospedale Maggiore | | | Bologna, Italy | | Local investigator | |  |
| Laura | Mastrangelo | | MD. | IRCCS Azienda Ospedaliera Universitaria di Bologna c/o Ospedale Maggiore | | | Bologna, Italy | | Local investigator | |  |
| Matteo | Rottoli | | MD. | IRCCS Azienda Ospedaliero-Universitaria di Bologna - Alma Mater Studiorum University of Bologna | | | Bologna, Italy | | Local investigator | |  |
| Claudio | Ricci | | MD. | IRCCS Azienda Ospedaliero-Universitaria di Bologna - Alma Mater Studiorum University of Bologna | | | Bologna, Italy | | Local investigator | |  |
| Iris S | Russo | | MD. | IRCCS Azienda Ospedaliero-Universitaria di Bologna - Alma Mater Studiorum University of Bologna | | | Bologna, Italy | | Local investigator | |  |
| Alberto | Aiolfi | | MD. | Istituto Clinico Sant'Ambrogio, University of Milan | | | Milan, Italy | | Local investigator | |  |
| Davide | Bona | | MD. | Istituto Clinico Sant'Ambrogio, University of Milan | | | Milan, Italy | | Local investigator | |  |
| Francesca | Lombardo | | MD. | Istituto Clinico Sant'Ambrogio, University of Milan | | | Milan, Italy | | Local investigator | |  |
| Pasquale | Cianci | | MD. | "Lorenzo Bonomo" Hospital, Andria | | | Andria, Italy | | Local investigator | |  |
| Mariagrazia | Sederino | | MD. | "Lorenzo Bonomo" Hospital, Andria | | | Andria, Italy | | Local investigator | |  |
| Roberto | Bini | | MD. | Niguarda Hospital, Trauma Team | | | Milan, Italy | | Local investigator | |  |
| Osvaldo | Chiara | | MD. | Niguarda Hospital, Trauma Team | | | Milan, Italy | | Local investigator | |  |
| Stefano PB | Cioffi | | MD. | Niguarda Hospital, Trauma Team | | | Milan, Italy | | Local investigator | |  |
| Alessio | Giordano | | MD. | Nuovo Ospedale “S.Stefano” Prato | | | Prato, Italy | | Local investigator | |  |
| Stefano | Cantafio | | MD. | Nuovo Ospedale “S.Stefano” Prato | | | Prato, Italy | | Local investigator | |  |
| Guido | Coretti | | MD. | Ospedale Buonconsiglio Fatebenefratelli di Napoli | | | Napoli, Italy | | Local investigator | |  |
| Edelweiss | Licitra | | MD. | Ospedale Civile di Vittorio Veneto | | | Vittorio Veneto, Italy | | Local investigator | |  |
| Grazia | Savino | | MD. | Ospedale Civile di Vittorio Veneto | | | Vittorio Veneto, Italy | | Local investigator | |  |
| Sergio | Grimaldi | | MD. | Ospedale Convenzionato Villa dei Fiori Acerra | | | Acerra, Italy | | Local investigator | |  |
| Raffaele | Porfidia | | MD. | Ospedale Convenzionato Villa dei Fiori Acerra | | | Acerra, Italy | | Local investigator | |  |
| Elisabetta | Moggia | | MD. | Ospedale degli Infermi Rivoli Asl To 3 | | | Torino, Italy | | Local investigator | |  |
| Mauro | Garino | | MD. | Ospedale degli Infermi Rivoli Asl To 3 | | | Torino, Italy | | Local investigator | |  |
| Chiara | Marafante | | MD. | Ospedale degli Infermi Rivoli Asl To 3 | | | Torino, Italy | | Local investigator | |  |
| Antonio | Pesce | | MD. | Ospedale del Delta, Azienda USL of Ferrara | | | Ferrara, Italy | | Local investigator | |  |
| Nicolò | Fabbri | | MD. | Ospedale del Delta, Azienda USL of Ferrara | | | Ferrara, Italy | | Local investigator | |  |
| Carlo V | Feo | | MD. | Ospedale del Delta, Azienda USL of Ferrara | | | Ferrara, Italy | | Local investigator | |  |
| Ester | Marra | | MD. | Ospedale del Mare | | | Napoli, Italy | | Local investigator | |  |
| Carlo | Nagliati | | MD. | Ospedale di Gorizia-Monfalcone, ASUGI | | | Gorizia, Italy | | Local investigator | |  |
| Marina | Troian | | MD. | Ospedale di Gorizia-Monfalcone, ASUGI | | | Gorizia, Italy | | Local investigator | |  |
| Davide | Drigo | | MD. | Ospedale di Gorizia-Monfalcone, ASUGI | | | Gorizia, Italy | | Local investigator | |  |
| Andrea | Muratore | | MD. | Ospedale di Pinerolo | | | Torino, Italy | | Local investigator | |  |
| Riccardo | Danna | | MD. | Ospedale di Pinerolo | | | Torino, Italy | | Local investigator | |  |
| Alessandra | Murgese | | MD. | Ospedale di Pinerolo | | | Torino, Italy | | Local investigator | |  |
| Michele | Crespi | | MD. | ASST Fatebenefratelli Luigi Sacco, Milano | | | Milan, Italy | | Local investigator | |  |
| Luca | Ferrario | | MD. | ASST Fatebenefratelli Luigi Sacco, Milano | | | Milan, Italy | | Local investigator | |  |
| Claudio | Guerci | | MD. | ASST Fatebenefratelli Luigi Sacco, Milano | | | Milan, Italy | | Local investigator | |  |
| Alice | Frontali | | MD. | ASST Fatebenefratelli Luigi Sacco, Milano | | | Milan, Italy | | Local investigator | |  |
| Luca | Ferrari | | MD. | ASST Fatebenefratelli Luigi Sacco, Milano | | | Milan, Italy | | Local investigator | |  |
| Francesco | Favi | | MD. | Ospedale M.Bufalini Ausl della Romagna | | | Cesena, Italy | | Local investigator | |  |
| Erika | Picariello | | MD. | Ospedale M.Bufalini Ausl della Romagna | | | Cesena, Italy | | Local investigator | |  |
| Alessia | Rampini | | MD. | Ospedale M.Bufalini Ausl della Romagna | | | Cesena, Italy | | Local investigator | |  |
| Fabrizio | D'Acapito | | MD. | Ospedale Morgagni-Pierantoni AUSL Romagna Forlì | | | Forlì, Italy | | Local investigator | |  |
| Giorgio | Ercolani | | MD. | Ospedale Morgagni-Pierantoni AUSL Romagna Forlì | | | Forlì, Italy | | Local investigator | |  |
| Leonardo | Solaini | | MD. | Ospedale Morgagni-Pierantoni AUSL Romagna Forlì | | | Forlì, Italy | | Local investigator | |  |
| Francesco | Palmieri | | MD. | Ospedale San Carlo Borromeo | | | Milan, Italy | | Local investigator | |  |
| Matteo | Calì | | MD. | Ospedale San Carlo Borromeo | | | Milan, Italy | | Local investigator | |  |
| Francesco | Ferrara | | MD. | Ospedale San Carlo Borromeo | | | Milan, Italy | | Local investigator | |  |
| Irnerio A | Muttillo | | MD. | Ospedale San Filippo Neri, Roma | | | Roma, Italy | | Local investigator | |  |
| Edoardo M | Muttillo | | MD. | Ospedale San Filippo Neri, Roma | | | Roma, Italy | | Local investigator | |  |
| Biagio | Picardi | | MD. | Ospedale San Filippo Neri, Roma | | | Roma, Italy | | Local investigator | |  |
| Raffaele | Galleano | | MD. | Ospedale Santa Corona | | | Pietra Ligure, Italy | | Local investigator | |  |
| Ali | Badran | | MD. | Ospedale Santa Corona | | | Pietra Ligure, Italy | | Local investigator | |  |
| Omar | Ghazouani | | MD. | Ospedale Santa Corona | | | Pietra Ligure, Italy | | Local investigator | |  |
| Maurizio | Cervellera | | MD. | Ospedale SS Annunziata | | | Taranto, Italy | | Local investigator | |  |
| Gaetano | Campanella | | MD. | Ospedale SS Annunziata | | | Taranto, Italy | | Local investigator | |  |
| Gennaro | Papa | | MD. | Ospedale SS Annunziata | | | Taranto, Italy | | Local investigator | |  |
| Annamaria | Di Bella | | MD. | Ospedale SS. Cosma e Damiano, Pescia | | | Pescia, Italy | | Local investigator | |  |
| Gennaro | Perrone | | MD. | Parma University Hospital | | | Parma, Italy | | Local investigator | |  |
| Gabriele L | Petracca | | MD. | Parma University Hospital | | | Parma, Italy | | Local investigator | |  |
| Concetta | Prioriello | | MD. | Parma University Hospital | | | Parma, Italy | | Local investigator | |  |
| Mario | Giuffrida | | MD. | Parma University Hospital | | | Parma, Italy | | Local investigator | |  |
| Federico | Cozzani | | MD. | Parma University Hospital | | | Parma, Italy | | Local investigator | |  |
| Matteo | Rossini | | MD. | Parma University Hospital | | | Parma, Italy | | Local investigator | |  |
| Marco | Inama | | MD. | Pederzoli Hospital | | | Peschiera del Garda, Italy | | Local investigator | |  |
| Giovanni | Butturini | | MD. | Pederzoli Hospital | | | Peschiera del Garda, Italy | | Local investigator | |  |
| Gianluigi | Moretto | | MD. | Pederzoli Hospital | | | Peschiera del Garda, Italy | | Local investigator | |  |
| Luca | Morelli | | MD. | Pisa University; Department of Translational Research and new Technologies in Medicine and Surgery | | | Pisa, Italy | | Local investigator | |  |
| Giulio | Di Candio | | MD. | Pisa University; Department of Translational Research and new Technologies in Medicine and Surgery | | | Pisa, Italy | | Local investigator | |  |
| Simone | Guadagni | | MD. | Pisa University; Department of Translational Research and new Technologies in Medicine and Surgery | | | Pisa, Italy | | Local investigator | |  |
| Enrico | Cicuttin | | MD. | Pisa University Hospital, General, Emergency and Trauma Surgery, Pisa | | | Pisa, Italy | | Local investigator | |  |
| Camilla | Cremonini | | MD. | Pisa University Hospital, General, Emergency and Trauma Surgery, Pisa | | | Pisa, Italy | | Local investigator | |  |
| Dario | Tartaglia | | MD. | Pisa University Hospital, General, Emergency and Trauma Surgery, Pisa | | | Pisa, Italy | | Local investigator | |  |
| Valerio | Genovese | | MD. | Pisa University Hospital, General, Emergency and Trauma Surgery, Pisa | | | Pisa, Italy | | Local investigator | |  |
| Massimo | Chiarugi | | MD. | Pisa University Hospital, General, Emergency and Trauma Surgery, Pisa | | | Pisa, Italy | | Local investigator | |  |
| Nicola | Cillara | | MD. | PO SS. Trinità ASSL Cagliari ATS Sardegna | | | Cagliari, Italy | | Local investigator | |  |
| Alessandro | Cannavera | | MD. | PO SS. Trinità ASSL Cagliari ATS Sardegna | | | Cagliari, Italy | | Local investigator | |  |
| Antonello | Deserra | | MD. | PO SS. Trinità ASSL Cagliari ATS Sardegna | | | Cagliari, Italy | | Local investigator | |  |
| Arcangelo | Picciariello | | MD. | Policlinico di Bari | | | Bari, Italy | | Local investigator | |  |
| Vincenzo | Papagni | | MD. | Policlinico di Bari | | | Bari, Italy | | Local investigator | |  |
| Leonardo | Vincenti | | MD. | Policlinico di Bari | | | Bari, Italy | | Local investigator | |  |
| Giulia | Bagaglini | | MD. | Policlinico di Roma Tor Vergata | | | Roma, Italy | | Local investigator | |  |
| Giuseppe | Sica | | MD. | Policlinico di Roma Tor Vergata | | | Roma, Italy | | Local investigator | |  |
| Pierfrancesco | Lapolla | | MD. | Policlinico Umberto I Sapienza University of Rome | | | Roma, Italy | | Local investigator | |  |
| Gioia | Brachini | | MD. | Policlinico Umberto I Sapienza University of Rome | | | Roma, Italy | | Local investigator | |  |
| Andrea | Mingoli | | MD. | Policlinico Umberto I Sapienza University of Rome | | | Roma, Italy | | Local investigator | |  |
| Dario | Bono | | MD. | Presidio Ospedaliero Santi Pietro e Paolo Borgosesia | | | Borgosesia, Italy | | Local investigator | |  |
| Antonella | Nicotera | | MD. | Presidio Ospedaliero Santi Pietro e Paolo Borgosesia | | | Borgosesia, Italy | | Local investigator | |  |
| Marcello | Zago | | MD. | Presidio Ospedaliero Santi Pietro e Paolo Borgosesia | | | Borgosesia, Italy | | Local investigator | |  |
| Fabrizio | Sammartano | | MD. | San Carlo Borromeo Hospital | | | Milan, Italy | | Local investigator | |  |
| Laura | Benuzzi | | MD. | San Carlo Borromeo Hospital | | | Milan, Italy | | Local investigator | |  |
| Marco | Stella | | MD. | San Carlo Borromeo Hospital | | | Milan, Italy | | Local investigator | |  |
| Stefano | Rossi | | MD. | San Filippo Neri Hospital | | | Roma, Italy | | Local investigator | |  |
| Alessandra | Cerioli | | MD. | San Filippo Neri Hospital | | | Roma, Italy | | Local investigator | |  |
| Caterina | Puccioni | | MD. | San Filippo Neri Hospital | | | Roma, Italy | | Local investigator | |  |
| Stefano | Olmi | | MD. | San Marco Hospital GSD – Zingonia | | | Bergamo, Italy | | Local investigator | |  |
| Carolina | Rubicondo | | MD. | San Marco Hospital GSD – Zingonia | | | Bergamo, Italy | | Local investigator | |  |
| Matteo | Uccelli | | MD. | San Marco Hospital GSD – Zingonia | | | Bergamo, Italy | | Local investigator | |  |
| Andrea | Balla | | MD. | San Paolo Hospital Civitavecchia | | | Civitavecchia, Italy | | Local investigator | |  |
| Anna | Guida | | MD. | San Paolo Hospital Civitavecchia | | | Civitavecchia, Italy | | Local investigator | |  |
| Pasquale | Lepiane | | MD. | San Paolo Hospital Civitavecchia | | | Civitavecchia, Italy | | Local investigator | |  |
| Diego | Sasia | | MD. | Santa Croce and Carle Hospital | | | Cuneo, Italy | | Local investigator | |  |
| Giorgio | Giraudo | | MD. | Santa Croce and Carle Hospital | | | Cuneo, Italy | | Local investigator | |  |
| Sara | Salomone | | MD. | Santa Croce and Carle Hospital | | | Cuneo, Italy | | Local investigator | |  |
| Giuseppe | Nigri | | MD. | Sapienza University of Rome, Sant’Andrea Hospital | | | Roma, Italy | | Local investigator | |  |
| Elena | Belloni | | MD. | Sapienza University of Rome, Sant’Andrea Hospital | | | Roma, Italy | | Local investigator | |  |
| Alessandra | Cossa | | MD. | Sapienza University of Rome, Sant’Andrea Hospital | | | Roma, Italy | | Local investigator | |  |
| Francesco | Lancellotti | | MD. | Sapienza University of Rome | | | Roma, Italy | | Local investigator | |  |
| Roberto | Caronna | | MD. | Sapienza University of Rome | | | Roma, Italy | | Local investigator | |  |
| Piero | Chirletti | | MD. | Sapienza University of Rome | | | Roma, Italy | | Local investigator | |  |
| Paolina | Saullo | | MD. | Sapienza University of Rome | | | Roma, Italy | | Local investigator | |  |
| Raffaele | Troiano | | MD. | Sapienza University of Rome | | | Roma, Italy | | Local investigator | |  |
| Felice | Mucilli | | MD. | SS. Annunziata Hospital of Chieti | | | Chieti, Italy | | Local investigator | |  |
| Mirko | Barone | | MD. | SS. Annunziata Hospital of Chieti | | | Chieti, Italy | | Local investigator | |  |
| Massimo | Ippoliti | | MD. | SS. Annunziata Hospital of Chieti | | | Chieti, Italy | | Local investigator | |  |
| Michele | Grande | | MD. | Università degli Studi di Roma "Tor Vergata" | | | Roma, Italy | | Local investigator | |  |
| Bruno | Sensi | | MD. | Università degli Studi di Roma "Tor Vergata" | | | Roma, Italy | | Local investigator | |  |
| Leandro | Siragusa | | MD. | Università degli Studi di Roma "Tor Vergata" | | | Roma, Italy | | Local investigator | |  |
| Monica | Ortenzi | | MD. | Università Politecnica delle Marche | | | Ancona, Italy | | Local investigator | |  |
| Andrea | Santini | | MD. | Università Politecnica delle Marche | | | Ancona, Italy | | Local investigator | |  |
| Gastone | Veroux | | MD. | University of Catania | | | Catania, Italy | | Local investigator | |  |
| Isidoro | Di Carlo | | MD. | University of Catania | | | Catania, Italy | | Local investigator | |  |
| Rossella | Gioco | | MD. | University of Catania | | | Catania, Italy | | Local investigator | |  |
| Massimiliano | Veroux | | MD. | University of Catania | | | Catania, Italy | | Local investigator | |  |
| Giuseppe | Currò | | MD. | "Magna Graecia" University Medical School of Catanzaro | | | Catanzaro, Italy | | Local investigator | |  |
| Michele | Ammendola | | MD. | "Magna Graecia" University Medical School of Catanzaro | | | Catanzaro, Italy | | Local investigator | |  |
| Iman | Komaei | | MD. | University Hospital of Messina | | | Messina, Italy | | Local investigator | |  |
| Giuseppe | Navarra | | MD. | University Hospital of Messina | | | Messina, Italy | | Local investigator | |  |
| Valeria | Tonini | | MD. | University of Bologna, General Surgery | | | Bologna, Italy | | Local investigator | |  |
| Lodovico | Sartarelli | | MD. | University of Bologna, General Surgery | | | Bologna, Italy | | Local investigator | |  |
| Samuele | Vaccari | | MD. | University of Bologna, General Surgery | | | Bologna, Italy | | Local investigator | |  |
| Marco | Ceresoli | | MD. | University of Milano-Bicocca | | | Milan, Italy | | Local investigator | |  |
| Stefano | Perrone | | MD. | University of Milano-Bicocca | | | Milan, Italy | | Local investigator | |  |
| Linda | Roccamatisi | | MD. | University of Milano-Bicocca | | | Milan, Italy | | Local investigator | |  |
| Paolo | Millo | | MD. | "U. Parini" Regional Hospital | | | Aosta, Italy | | Local investigator | |  |
| Riccardo | Brachet-Contul | | MD. | "U. Parini" Regional Hospital | | | Aosta, Italy | | Local investigator | |  |
| Elisa | Ponte | | MD. | "U. Parini" Regional Hospital | | | Aosta, Italy | | Local investigator | |  |
| Matteo | Zuin | | MD. | U.O.C. Chirurgia Generale, Ospedale di Cittadella - ULSS 6 Euganea | | | Cittadella, Italy | | Local investigator | |  |
| Giuseppe | Portale | | MD. | U.O.C. Chirurgia Generale, Ospedale di Cittadella - ULSS 6 Euganea | | | Cittadella, Italy | | Local investigator | |  |
| Alice S | Tonello | | MD. | U.O.C. Chirurgia Generale, Ospedale di Cittadella - ULSS 6 Euganea | | | Cittadella, Italy | | Local investigator | |  |
| Geri | Fratini | | MD. | Versilia Hospital Toscana nordovest | | | Lido di Camaiore, Italy | | Local investigator | |  |
| Matteo | Bianchini | | MD. | Versilia Hospital Toscana nordovest | | | Lido di Camaiore, Italy | | Local investigator | |  |
| Bruno | Perotti | | MD. | Versilia Hospital Toscana nordovest | | | Lido di Camaiore, Italy | | Local investigator | |  |
| Emanuele | Doria | | MD. | AOU Città della Salute e della Scienza | | | Torino, Italy | | Local investigator | |  |
| Elia G | Lunghi | | MD. | AOU Città della Salute e della Scienza | | | Torino, Italy | | Local investigator | |  |
| Diego | Visconti | | MD. | AOU Città della Salute e della Scienza | | | Torino, Italy | | Local investigator | |  |
| Almu'atasim | Khamees | | MD. | Faculty of Medicine, Yarmouk University | | | Yarmouk, Jordan | | Local investigator | |  |
| Khayry | Al-Shami | | MD. | Faculty of Medicine, Yarmouk University | | | Yarmouk, Jordan | | Local investigator | |  |
| Sajeda | Awadi | | MD. | Faculty of Medicine, Yarmouk University | | | Yarmouk, Jordan | | Local investigator | |  |
| Hazim | Ababneh | | MD. | King Hussein Medical Center-Royal Medical Services | | | Amman, Jordan | | Local investigator | |  |
| Mohammad MK | Buwaitel | | MD. | King Hussein Medical Center-Royal Medical Services | | | Amman, Jordan | | Local investigator | |  |
| Mo'taz FN | Naffa' | | MD. | King Hussein Medical Center-Royal Medical Services | | | Amman, Jordan | | Local investigator | |  |
| Osama | Aljaiuossi | | MD. | Prince Faisal Hospital - Ministry of Health | | | Zarqa, Jordan | | Local investigator | |  |
| Ahmad | Samhouri | | MD. | Prince Faisal Hospital - Ministry of Health | | | Zarqa, Jordan | | Local investigator | |  |
| Hatem | Sawalha | | MD. | Prince Faisal Hospital - Ministry of Health | | | Zarqa, Jordan | | Local investigator | |  |
| Ahmad R | Yusoff | | MD. | Faculty of Medicine, Universiti Teknologi MARA | | | Selangor, Malaysia | | Local investigator | |  |
| Mohd F | Che Ani | | MD. | Faculty of Medicine, Universiti Teknologi MARA | | | Selangor, Malaysia | | Local investigator | |  |
| Ida NA | Fathil | | MD. | Faculty of Medicine, Universiti Teknologi MARA | | | Selangor, Malaysia | | Local investigator | |  |
| Jih | Huei | | MD. | Hospital Sultanah Aminah | | | Johor Bahru, Malaysia | | Local investigator | |  |
| Ikhwan S | Mohamad | | MD. | Hospital University Sains Malaysia | | | Kelantan, Malaysia | | Local investigator | |  |
| Andee D | Zakaria | | MD. | Hospital University Sains Malaysia | | | Kelantan, Malaysia | | Local investigator | |  |
| Mohammad Z | Ya'acob | | MD. | Hospital University Sains Malaysia | | | Kelantan, Malaysia | | Local investigator | |  |
| Jose L | Beristain-Hernandez | | MD. | Hospital de Especialidades "Antonio Fraga Mouret". Centro Medico Nacional "La Raza". Instituto Mexicano del Seguro Social | | | Ciudad de Mexico, Mexico | | Local investigator | |  |
| Alejandro | Garcia-Meza | | MD. | Hospital de Especialidades "Antonio Fraga Mouret". Centro Medico Nacional "La Raza". Instituto Mexicano del Seguro Social | | | Ciudad de Mexico, Mexico | | Local investigator | |  |
| Rafael | Sepulveda-Rodriguez | | MD. | Hospital de Especialidades "Antonio Fraga Mouret". Centro Medico Nacional "La Raza". Instituto Mexicano del Seguro Social | | | Ciudad de Mexico, Mexico | | Local investigator | |  |
| Edgard E | Lozada Hernández | | MD. | Hospital Regional de Alta Especialidad del Bajío | | | Leon, Mexico | | Local investigator | |  |
| Camilo L | Acuña Pinzón | | MD. | Hospital Regional de Alta Especialidad del Bajío | | | Leon, Mexico | | Local investigator | |  |
| Jefferson N | Condoy | | MD. | Hospital Regional de Alta Especialidad del Bajío | | | Leon, Mexico | | Local investigator | |  |
| Francisco C | Becerra García | | MD. | Hospital San Ángel Inn Patriotismo | | | Ciudad de Mexico, Mexico | | Local investigator | |  |
| Samuel | Pimentel | | MD. | Imss General Regional Hospital | | | Ciudad de Mexico, Mexico | | Local investigator | |  |
| Ibrahim U | Garzali | | MD. | Aminu Kano Teaching Hospital | | | Kano, Nigeria | | Local investigator | |  |
| Mohammad | Sadik | | MD. | Asian Institute of Medical Sciences | | | Hyderabad, Pakistan | | Local investigator | |  |
| Bushra | Kadir | | MD. | Asian Institute of Medical Sciences | | | Hyderabad, Pakistan | | Local investigator | |  |
| Jalpa | Devi | | MD. | Liaquat University of Medical and Health Sciences | | | Sindh, Pakistan | | Local investigator | |  |
| Nandlal | Seerani | | MD. | Liaquat University of Medical and Health Sciences | | | Sindh, Pakistan | | Local investigator | |  |
| Mohammad | Sohail- Asghar | | MD. | Mayo Hospital King Edward Medical University Lahore | | | Lahore, Pakistan | | Local investigator | |  |
| Ameer | Afzal | | MD. | Mayo Hospital King Edward Medical University Lahore | | | Lahore, Pakistan | | Local investigator | |  |
| Ali | Akbar | | MD. | Mayo Hospital King Edward Medical University Lahore | | | Lahore, Pakistan | | Local investigator | |  |
| Gustavo M | Machain | | MD. | Universidad Nacional de Asunción- Facultad de Ciencias Medicas. Hospital de Clinicas | | | San Lorenzo, Paraguay | | Local investigator | |  |
| Helmut | Segovia Lohse | | MD. | Universidad Nacional de Asunción- Facultad de Ciencias Medicas. Hospital de Clinicas | | | San Lorenzo, Paraguay | | Local investigator | |  |
| Herald | Segovia Lohse | | MD. | Universidad Nacional de Asunción- Facultad de Ciencias Medicas. Hospital de Clinicas | | | San Lorenzo, Paraguay | | Local investigator | |  |
| Darwin A | Quispe-Cruz | | MD. | Cayetano Heredia National Hospital – Lima | | | Lima, Peru | | Local investigator | |  |
| Zamiara SL | Cabrera | | MD. | Cayetano Heredia National Hospital – Lima | | | Lima, Peru | | Local investigator | |  |
| Gaby S | Yamamoto Seto | | MD. | Cayetano Heredia National Hospital – Lima | | | Lima, Peru | | Local investigator | |  |
| José R | Chiuyari | | MD. | Universidad Nacional Pedro Ruiz Gallo Facultad de Medicina Humana, Universidad Peruana Los Andes | | | Huancayo, Peru | | Local investigator | |  |
| Jorge | Ordemar | | MD. | Universidad Nacional Pedro Ruiz Gallo Facultad de Medicina Humana, Universidad Peruana Los Andes | | | Huancayo, Peru | | Local investigator | |  |
| Martha | Rodríguez | | MD. | Universidad Nacional Pedro Ruiz Gallo Facultad de Medicina Humana, Universidad Peruana Los Andes | | | Huancayo, Peru | | Local investigator | |  |
| Abigail CC | Orantia-Carlos | | MD. | Medical Center Manila- ManilaMed | | | Manila, Philippines | | Local investigator | |  |
| Margie A | Quitoy | | MD. | Medical Center Manila- ManilaMed | | | Manila, Philippines | | Local investigator | |  |
| Maciej | Walędziak | | MD. | Military Institute of Medicine | | | Warsaw, Poland | | Local investigator | |  |
| Andrzej | Kwiatkowski | | MD. | Military Institute of Medicine | | | Warsaw, Poland | | Local investigator | |  |
| Maciej | Mawlichanów | | MD. | Military Institute of Medicine | | | Warsaw, Poland | | Local investigator | |  |
| Tiago | Correia de Sá | | MD. | Centro Hospitalar do Tâmega e Sousa | | | Guilhufe, Portugal | | Local investigator | |  |
| Mónica | Rocha | | MD. | Centro Hospitalar do Tâmega e Sousa | | | Guilhufe, Portugal | | Local investigator | |  |
| Carlos | Soares | | MD. | Centro Hospitalar do Tâmega e Sousa | | | Guilhufe, Portugal | | Local investigator | |  |
| Syed | Muhammad Ali | | MD. | Hamad Medical Corporation | | | Doha, Quatar | | Local investigator | |  |
| Alexandru R | Stoian | | MD. | "Bagdasar-Arseni" Clinical Emergency Hospital | | | Bucharest, Romania | | Local investigator | |  |
| Andreea D | Draghici | | MD. | "Bagdasar-Arseni" Clinical Emergency Hospital | | | Bucharest, Romania | | Local investigator | |  |
| Valentin T | Grigorean | | MD. | "Bagdasar-Arseni" Clinical Emergency Hospital | | | Bucharest, Romania | | Local investigator | |  |
| Valentin | Calu | | MD. | Elias Emergency University Hospital | | | Bucharest, Romania | | Local investigator | |  |
| Raluca B | Radulescu | | MD. | Spitalul Clinic De Urgenta | | | Bucharest, Romania | | Local investigator | |  |
| Narcis O | Zarnescu | | MD. | University Emergency Hospital Bucharest, "Carol Davila", University of Medicine and Pharmacy Bucharest | | | Bucharest, Romania | | Local investigator | |  |
| Radu V | Costea | | MD. | University Emergency Hospital Bucharest, "Carol Davila", University of Medicine and Pharmacy Bucharest | | | Bucharest, Romania | | Local investigator | |  |
| Eugenia C | Zarnescu | | MD. | University Emergency Hospital Bucharest, "Carol Davila", University of Medicine and Pharmacy Bucharest | | | Bucharest, Romania | | Local investigator | |  |
| Andrey | Litvin | | MD. | Immanuel Kant Baltic Federal University, Regional Clinical Hospital | | | Kaliningrad, Russia | | Local investigator | |  |
| Mikhail | Kurtenkov | | MD. | Immanuel Kant Baltic Federal University, Regional Clinical Hospital | | | Kaliningrad, Russia | | Local investigator | |  |
| George | Gendrikson | | MD. | Immanuel Kant Baltic Federal University, Regional Clinical Hospital | | | Kaliningrad, Russia | | Local investigator | |  |
| Volovich | Alla-Angelina | | MD. | Immanuel Kant Baltic Federal University, Regional Clinical Hospital | | | Kaliningrad, Russia | | Local investigator | |  |
| Arina | Tsurbanova | | MD. | Immanuel Kant Baltic Federal University, Regional Clinical Hospital | | | Kaliningrad, Russia | | Local investigator | |  |
| Ayrat | Kaldarov | | MD. | Vishnevsky Center of Surgery | | | Moscow, Russia | | Local investigator | |  |
| Mahir | Gachabayov | | MD. | Vladimir City Emergency Hospital | | | Vladimir City, Russia | | Local investigator | |  |
| Abakar | Abdullaev | | MD. | Vladimir City Emergency Hospital | | | Vladimir City, Russia | | Local investigator | |  |
| Bojan | Kovacevic | | MD. | Univesity Medical Center Zvezdara | | | Belgrade, Serbia | | Local investigator | |  |
| Milica | Milentijevic | | MD. | Univesity Medical Center Zvezdara | | | Belgrade, Serbia | | Local investigator | |  |
| Milovan | Karamarkovic | | MD. | Univesity Medical Center Zvezdara | | | Belgrade, Serbia | | Local investigator | |  |
| Arpád | Panyko | | MD. | University Hospital Bratislava | | | Bratislava, Slovak Republic | | Local investigator | |  |
| Dusan | Lesko | | MD. | University Hospital L. Pasteur | | | Kosice, Slovak Republic | | Local investigator | |  |
| Jozef | Radonak | | MD. | University Hospital L. Pasteur | | | Kosice, Slovak Republic | | Local investigator | |  |
| Marek | Soltes | | MD. | University Hospital L. Pasteur | | | Kosice, Slovak Republic | | Local investigator | |  |
| Colin | Noel | | MD. | Universitas Academic Hospital | | | Bloemfontein, South Africa | | Local investigator | |  |
| Haidar M | Abdalah | | MD. | AlSaaha Specialized Hospital | | | Al Khartoum, Sudan | | Local investigator | |  |
| Hytham KS | Hamid | | MD. | AlSaaha Specialized Hospital | | | Al Khartoum, Sudan | | Local investigator | |  |
| Raffaello | Roesel | | MD. | EOC Regional Hospital of Lugano | | | Lugano, Switzerland | | Local investigator | |  |
| Alessandra | Cristaudi | | MD. | EOC Regional Hospital of Lugano | | | Lugano, Switzerland | | Local investigator | |  |
| Cristiana | Riboni | | MD. | EOC Regional Hospital of Lugano | | | Lugano, Switzerland | | Local investigator | |  |
| Alaa | Hamdan | | MD. | Tishreen University Hopital | | | Latakya, Syria | | Local investigator | |  |
| Kinan | Abbas | | MD. | Tishreen University Hopital | | | Latakya, Syria | | Local investigator | |  |
| Iyad | Ali | | MD. | Tishreen University Hopital | | | Latakya, Syria | | Local investigator | |  |
| Ali | Kchaou | | MD. | Habib Bourguiba University Hospital | | | Sfax, Tunisia | | Local investigator | |  |
| Ahmed | Tlili | | MD. | Habib Bourguiba University Hospital | | | Sfax, Tunisia | | Local investigator | |  |
| Arda | Isik | | MD. | Erzincan University | | | Erzinkan, Turkey | | Local investigator | |  |
| Hüseyin | Bayhan | | MD. | Mardin Training and Research Hospital | | | Mardin, Turkey | | Local investigator | |  |
| Mehmet A | Türkoğlu | | MD. | Mardin Training and Research Hospital | | | Mardin, Turkey | | Local investigator | |  |
| Mustafa Y | Uzunoglu | | MD. | Kestel State Hospital | | | Kestel, Turkey | | Local investigator | |  |
| Ibrahim F | Azamat | | MD. | Koç University Hospital | | | Koç, Turkey | | Local investigator | |  |
| Nail | Omarov | | MD. | Koç University Hospital | | | Koç, Turkey | | Local investigator | |  |
| Derya S | Uymaz | | MD. | Koç University Hospital | | | Koç, Turkey | | Local investigator | |  |
| Fatih | Altintoprak | | MD. | Sakarya University Faculty of Medicine | | | Sakarya, Turkey | | Local investigator | |  |
| Emrah | Akin | | MD. | Sakarya University Faculty of Medicine | | | Sakarya, Turkey | | Local investigator | |  |
| Necattin | First | | MD. | Sakarya University Faculty of Medicine | | | Sakarya, Turkey | | Local investigator | |  |
| Koray | Das | | MD. | University of Health Sciences, Adana City Training and Research Hospital | | | Adana, Turkey | | Local investigator | |  |
| Nazmi | Ozer | | MD. | University of Health Sciences, Adana City Training and Research Hospital | | | Adana, Turkey | | Local investigator | |  |
| Ahmet | Seker | | MD. | University of Health Sciences, Adana City Training and Research Hospital | | | Adana, Turkey | | Local investigator | |  |
| Yasin | Kara | | MD. | Health Sciences University, Kanuni Sultan Süleyman Training and Research Hospital | | | Istanbul, Turkey | | Local investigator | |  |
| Mehmet A | Bozkurt | | MD. | Health Sciences University, Kanuni Sultan Süleyman Training and Research Hospital | | | Istanbul, Turkey | | Local investigator | |  |
| Ali | Kocataş | | MD. | Health Sciences University, Kanuni Sultan Süleyman Training and Research Hospital | | | Istanbul, Turkey | | Local investigator | |  |
| Semra D | Atici | | MD. | University of Health Sciences Tepecik Training and Research Hospital, Department of General Surgery | | | Izmir, Turkey | | Local investigator | |  |
| Murat | Akalin | | MD. | University of Health Sciences Tepecik Training and Research Hospital, Department of General Surgery | | | Izmir, Turkey | | Local investigator | |  |
| Bulent | Calik | | MD. | University of Health Sciences Tepecik Training and Research Hospital, Department of General Surgery | | | Izmir, Turkey | | Local investigator | |  |
| Elif | Colak | | MD. | University of Health Sciences Turkey, Samsun Training and Research Hospital | | | Izmir, Turkey | | Local investigator | |  |
| Yuksel | Altinel | | MD. | Bagcilar Research and Training Hospital | | | Istanbul, Turkey | | Local investigator | |  |
| Serhat | Meric | | MD. | Bagcilar Research and Training Hospital | | | Istanbul, Turkey | | Local investigator | |  |
| Yunus E | Aktimur | | MD. | Bagcilar Research and Training Hospital | | | Istanbul, Turkey | | Local investigator | |  |
| Serge | Chooklin | | MD. | Lviv Regional Clinical Hospital | | | Lviv, Ukraine | | Local investigator | |  |
| Serhii | Chuklin | | MD. | Lviv Regional Clinical Hospital | | | Lviv, Ukraine | | Local investigator | |  |
| Andriy | Bilyak | | MD. | Lviv Regional Clinical Hospital | | | Lviv, Ukraine | | Local investigator | |  |
| Fernando | Bonilla Cal | | MD. | Hospital Español, Montevideo | | | Montevideo, Uruguay | | Local investigator | |  |
| Lianet | Sánchez | | MD. | Hospital Español, Montevideo | | | Montevideo, Uruguay | | Local investigator | |  |
| Fabiana | Domínguez | | MD. | Hospital Español, Montevideo | | | Montevideo, Uruguay | | Local investigator | |  |
| Abdullah | Meead | | MD. | Sana'a University Hospital | | | Sana'a, Yemen | | Local investigator | |  |
| Ibrahim | Al-Raimi | | MD. | Sana'a University Hospital | | | Sana'a, Yemen | | Local investigator | |  |
| Haneen | Alshargabi | | MD. | Sana'a University Hospital | | | Sana'a, Yemen | | Local investigator | |  |
| Victoria | Hudson | | MD. | Addenbrookes Hospital, Cambridge University Hospitals NHS Foundation Trust | | | Cambridge, United Kingdom | | Local investigator | |  |
| Jean-Luc | Duval | | MD. | Addenbrookes Hospital, Cambridge University Hospitals NHS Foundation Trust | | | Cambridge, United Kingdom | | Local investigator | |  |
| Stavros | Gourgiotis | | MD. | Addenbrookes Hospital, Cambridge University Hospitals NHS Foundation Trust | | | Cambridge, United Kingdom | | Local investigator | |  |
| Mansoor | Khan | | MD. | Brighton and Sussex University Hospital | | | Brighton, United Kingdom | | Local investigator | |  |
| Ahmed | Saad | | MD. | Brighton and Sussex University Hospital | | | Brighton, United Kingdom | | Local investigator | |  |
| Mandeep | Kaur | | MD. | Brighton and Sussex University Hospital | | | Brighton, United Kingdom | | Local investigator | |  |
| Michael | Wilson | | MD. | Forth Valley Royal Hospital | | | Larbert, United Kingdom | | Local investigator | |  |
| Alison | Bradley | | MD. | Forth Valley Royal Hospital | | | Larbert, United Kingdom | | Local investigator | |  |
| Katherine | Fox | | MD. | Forth Valley Royal Hospital | | | Larbert, United Kingdom | | Local investigator | |  |
| Ivan | Tomasi | | MD. | Guys and St Thomas Hospital London | | | London, United Kingdom | | Local investigator | |  |
| Daniel | Beasley | | MD. | Guys and St Thomas Hospital London | | | London, United Kingdom | | Local investigator | |  |
| Alekhya K | Prasanti | | MD. | Guys and St Thomas Hospital London | | | London, United Kingdom | | Local investigator | |  |
| Pinky | Kotecha | | MD. | Guys and St Thomas Hospital London | | | London, United Kingdom | | Local investigator | |  |
| Husam | Ebied | | MD. | Guys and St Thomas Hospital London | | | London, United Kingdom | | Local investigator | |  |
| Fiammetta | Soggiu | | MD. | London North West University Healthcare NHS Trust | | | London, United Kingdom | | Local investigator | |  |
| Michaela | Paul | | MD. | London North West University Healthcare NHS Trust | | | London, United Kingdom | | Local investigator | |  |
| Hemant | Sheth | | MD. | London North West University Healthcare NHS Trust | | | London, United Kingdom | | Local investigator | |  |
| Ioannis | Gerogiannis | | MD. | Kingston Hospital NHS Foundation Trust | | | Kingston, United Kingdom | | Local investigator | |  |
| Mohannad | Gaber | | MD. | Kingston Hospital NHS Foundation Trust | | | Kingston, United Kingdom | | Local investigator | |  |
| Zara | Sheikh | | MD. | Kingston Hospital NHS Foundation Trust | | | Kingston, United Kingdom | | Local investigator | |  |
| Shatadru | Seth | | MD. | Kingston Hospital NHS Foundation Trust | | | Kingston, United Kingdom | | Local investigator | |  |
| Maria | Kunitsyna | | MD. | Kingston Hospital NHS Foundation Trust | | | Kingston, United Kingdom | | Local investigator | |  |
| Cosimo A | Leo | | MD. | Northwick Park & St Mark's Hospital - London North West NHS Trust | | | London, United Kingdom | | Local investigator | |  |
| Vittoria | Bellato | | MD. | Northwick Park & St Mark's Hospital - London North West NHS Trust | | | London, United Kingdom | | Local investigator | |  |
| Noman | Zafar | | MD. | Northwick Park & St Mark's Hospital - London North West NHS Trust | | | London, United Kingdom | | Local investigator | |  |
| Amr | Elserafy | | MD. | Oxford University Hospitals NHS Foundation Trust | | | Oxford, United Kingdom | | Local investigator | |  |
| Giles | Bond-Smith | | MD. | Oxford University Hospitals NHS Foundation Trust | | | Oxford, United Kingdom | | Local investigator | |  |
| Giovanni | Tebala | | MD. | Oxford University Hospitals NHS Foundation Trust | | | Oxford, United Kingdom | | Local investigator | |  |
| Pawan | Mathur | | MD. | Royal Free London NHS Foundation Trust | | | London, United Kingdom | | Local investigator | |  |
| Izza | Abid | | MD. | Royal Free London NHS Foundation Trust | | | London, United Kingdom | | Local investigator | |  |
| Manuel | Abradelo | | MD. | University Hospitals Birmingham. Birmingham Heartlands Hospital | | | Birmingham, United Kingdom | | Local investigator | |  |
| Nnaemeka | Chidumije | | MD. | University Hospitals Birmingham. Birmingham Heartlands Hospital | | | Birmingham, United Kingdom | | Local investigator | |  |
| Pardip | Sandhar | | MD. | University Hospitals Birmingham. Birmingham Heartlands Hospital | | | Birmingham, United Kingdom | | Local investigator | |  |
| Syed OZ | Ullah | | MD. | University Hospitals Birmingham. Birmingham Heartlands Hospital | | | Birmingham, United Kingdom | | Local investigator | |  |
| Tamara | Lezama | | MD. | University Hospitals Birmingham. Birmingham Heartlands Hospital | | | Birmingham, United Kingdom | | Local investigator | |  |
| Muhammad H | Anwaar | | MD. | University Hospitals Birmingham. Birmingham Heartlands Hospital | | | Birmingham, United Kingdom | | Local investigator | |  |
| Conor | Magee | | MD. | Wirral University Teaching Hospitals NHS Foundation Trust | | | Birkenhead, United Kingdom | | Local investigator | |  |
| Salma | Ahmed | | MD. | Wirral University Teaching Hospitals NHS Foundation Trust | | | Birkenhead, United Kingdom | | Local investigator | |  |
| Brooke | Davies | | MD. | Wirral University Teaching Hospitals NHS Foundation Trust | | | Birkenhead, United Kingdom | | Local investigator | |  |
| Jeyakumar | Apollos | | MD. | Dumfries and Galloway Royal Infirmary | | | Cargenbridge, United Kingdom | | Local investigator | |  |
| Kieran | McCormack | | MD. | Dumfries and Galloway Royal Infirmary | | | Cargenbridge, United Kingdom | | Local investigator | |  |
| Hasham | Choudhary | | MD. | Dumfries and Galloway Royal Infirmary | | | Cargenbridge, United Kingdom | | Local investigator | |  |
| Triantafyllos | Doulias | | MD. | Colchester Hospital University NHS Foundation Trust | | | Colchester, United Kingdom | | Local investigator | |  |
| Tamsin | Morrison | | MD. | Colchester Hospital University NHS Foundation Trust | | | Colchester, United Kingdom | | Local investigator | |  |
| Anna | Palepa | | MD. | Colchester Hospital University NHS Foundation Trust | | | Colchester, United Kingdom | | Local investigator | |  |
| Laura | Álvarez Morán | | MD. | Complejo Asistencial Universitario de León | | | Leon, Spain | | Local investigator | |  |
| Haydée | Calvo García | | MD. | Complejo Asistencial Universitario de León | | | Leon, Spain | | Local investigator | |  |
| Pilar | Suárez Vega | | MD. | Complejo Asistencial Universitario de León | | | Leon, Spain | | Local investigator | |  |
| Sergio | Estevez | | MD. | Complejo Hospitalario Universitario de Vigo. Hospital Álvaro Cunqueiro | | | Vigo, Spain | | Local investigator | |  |
| Mikel | Prieto Calvo | | MD. | Cruces university Hospital, University of the Basque country | | | Bilbao, Spain | | Local investigator | |  |
| Ibabe | Villalabeitia | | MD. | Cruces university Hospital, University of the Basque country | | | Bilbao, Spain | | Local investigator | |  |
| Fabio | Ausania | | MD. | Hospital Clinic of Barcelona | | | Barcelona, Spain | | Local investigator | |  |
| Jordi | Farguell | | MD. | Hospital Clinic of Barcelona | | | Barcelona, Spain | | Local investigator | |  |
| Carolina | González-Abós | | MD. | Hospital Clinic of Barcelona | | | Barcelona, Spain | | Local investigator | |  |
| Santiago | Sánchez-Cabús | | MD. | Hospital de la Santa Creu i Sant Pau | | | Barcelona, Spain | | Local investigator | |  |
| Belén | Martín | | MD. | Hospital de la Santa Creu i Sant Pau | | | Barcelona, Spain | | Local investigator | |  |
| Víctor | Molina | | MD. | Hospital de la Santa Creu i Sant Pau | | | Barcelona, Spain | | Local investigator | |  |
| Luis | Oms | | MD. | Hospital de Terrassa | | | Barcelona, Spain | | Local investigator | |  |
| Lucas | Ilzarbe | | MD. | Hospital del Mar | | | Barcelona, Spain | | Local investigator | |  |
| Eva | Pont Feijóo | | MD. | Hospital del Mar | | | Barcelona, Spain | | Local investigator | |  |
| Elena S | Perra | | MD. | Hospital del Mar | | | Barcelona, Spain | | Local investigator | |  |
| Noel | Rojas-Bonet | | MD. | Hospital General d'Ontinyent | | | Valencia, Spain | | Local investigator | |  |
| Rafael | Penalba-Palmí | | MD. | Hospital General d'Ontinyent | | | Valencia, Spain | | Local investigator | |  |
| Susana | Pérez-Bru | | MD. | Hospital General d'Ontinyent | | | Valencia, Spain | | Local investigator | |  |
| Jaume | Tur-Martínez | | MD. | Hospital Universitario d'Igualada | | | Barcelona, Spain | | Local investigator | |  |
| Andrea | Álvarez-Torrado | | MD. | Hospital Universitario d'Igualada | | | Barcelona, Spain | | Local investigator | |  |
| Marta | Domingo-Gonzalez | | MD. | Hospital Universitario d'Igualada | | | Barcelona, Spain | | Local investigator | |  |
| Javier | Tejedor-Tejada | | MD. | Hospital Universitario de Cabueñes | | | Gijon, Spain | | Local investigator | |  |
| Yaiza | García del Alamo | | MD. | Hospital Universitario La Princesa | | | Madrid, Spain | | Local investigator | |  |
| Fernando | Mendoza-Moreno | | MD. | Hospital Universitario Príncipe de Asturias | | | Madrid, Spain | | Local investigator | |  |
| Francisca | García-Moreno-Nisa | | MD. | Hospital Universitario Príncipe de Asturias | | | Madrid, Spain | | Local investigator | |  |
| Belén | Matías-García | | MD. | Hospital Universitario Príncipe de Asturias | | | Madrid, Spain | | Local investigator | |  |
| Manuel | Durán | | MD. | Reina Sofía University Hospital | | | Cordoba, Spain | | Local investigator | |  |
| Rafael | Calleja-Lozano | | MD. | Reina Sofía University Hospital | | | Cordoba, Spain | | Local investigator | |  |
| José M | Perez de Villar | | MD. | Reina Sofía University Hospital | | | Cordoba, Spain | | Local investigator | |  |
| Luis | Sánchez-Guillén | | MD. | University General Hospital of Elche | | | Alicante, Spain | | Local investigator | |  |
| Iban | Caravaca | | MD. | University General Hospital of Elche | | | Alicante, Spain | | Local investigator | |  |
| Daniel | Triguero-Cánovas | | MD. | University General Hospital of Elche | | | Alicante, Spain | | Local investigator | |  |
| Antonio C | Maya Aparicio | | MD. | University Hospital Complex of Cáceres | | | Cáceres, Spain | | Local investigator | |  |
| Blas | Durán Meléndez | | MD. | University Hospital Complex of Cáceres | | | Cáceres, Spain | | Local investigator | |  |
| Andrea | Masiá Palacios | | MD. | University Hospital Complex of Cáceres | | | Cáceres, Spain | | Local investigator | |  |
| Aitor | Landaluce-Olavarria | | MD. | Urduliz Hospital | | | Urduliz, Spain | | Local investigator | |  |
| Mario | De Francisco | | MD. | Urduliz Hospital | | | Urduliz, Spain | | Local investigator | |  |
| Begoña | Estraviz-Mateos | | MD. | Urduliz Hospital | | | Urduliz, Spain | | Local investigator | |  |
| Felipe | Alconchel | | MD. | Virgen de la Arrixaca University Hospital, IMIB-Arrixaca | | | Murcia, Spain | | Local investigator | |  |
| Tatiana | Nicolás-López | | MD. | Virgen de la Arrixaca University Hospital, IMIB-Arrixaca | | | Murcia, Spain | | Local investigator | |  |
| Pablo | Ramírez | | MD. | Virgen de la Arrixaca University Hospital, IMIB-Arrixaca | | | Murcia, Spain | | Local investigator | |  |
| Virginia D | Muñoz-Cruzado | | MD. | Virgen del Rocío University Hospital | | | Seville, Spain | | Local investigator | |  |
| Felipe | Pareja Ciuró | | MD. | Virgen del Rocío University Hospital | | | Seville, Spain | | Local investigator | |  |
| Eduardo | Perea del Pozo | | MD. | Virgen del Rocío University Hospital | | | Seville, Spain | | Local investigator | |  |
| Sergio | Olivares Pizarro | | MD. | Infanta Cristina de Parla University Hospital | | | Madrid, Spain | | Local investigator | |  |
| Vicente | Herrera Cabrera | | MD. | Infanta Cristina de Parla University Hospital | | | Madrid, Spain | | Local investigator | |  |
| Jose | Muros Bayo | | MD. | Infanta Cristina de Parla University Hospital | | | Madrid, Spain | | Local investigator | |  |
