# Supplementary material for: Mild acute biliary pancreatitis: still a surgical disease. A post-hoc analysis of the MANCTRA-1 international study
Source: Eur J Trauma Emerg Surg. 2025 Jan 17;51(1):24. doi: 10.1007/s00068-024-02748-9 (PMC11742350; doi:10.1007/s00068-024-02748-9)
Supplement: Supplementary file 3 [file 68_2024_2748_MOESM3_ESM.docx]

|  | No 30-day readmission due to RAP  N = 1800 | 30-day readmission due to RAP  N = 120 | p-Value |
| --- | --- | --- | --- |
| **Patient age** | 59.43 (± 17.74) Range: (14.0 ; 96.0) N = 1800 | 56.02 (± 19.01) Range: (18.0 ; 92.0) N = 120 | 0.065 |
| **Sex** Female Male | 909 (50.5%) 891 (49.5%) N = 1800 | 61 (50.83%) 59 (49.17%) N = 120 | >0.999 |
| **Covid-19 status on admission** Negative Positive Untested | 886 (49.22%) 22 (1.22%) 892 (49.56%) N = 1800 | 50 (41.67%) 5 (4.17%) 65 (54.17%) N = 120 | **0.013** |
| **Previous episodes of biliary pancreatitis** No Not known Yes | 1355 (75.28%) 125 (6.94%) 320 (17.78%) N = 1800 | 93 (77.5%) 5 (4.17%) 22 (18.33%) N = 120 | 0.503 |
| **Admitting speciality** Med Surg | 958 (53.22%) 842 (46.78%) N = 1800 | 77 (64.17%) 43 (35.83%) N = 120 | **0.025** |
| **Setting of acquisition of the pancreatitis** Community acquired Hospital acquired | 1728 (96.0%) 72 (4.0%) N = 1800 | 115 (95.83%) 5 (4.17%) N = 120 | 0.812 |
| **Charlson's comorbidity index - available at: https://www.mdcalc.com/charlson-comorbidity-index-cci** | 2.68 (± 4.13) Range: (0.0 ; 98.0) N = 1800 | 2.24 (± 2.33) Range: (0.0 ; 15.0) N = 120 | 0.283 |
| **Body Mass Index - BMI (Kg/m2)** | 27.54 (± 5.28) Range: (0.0 ; 52.5) N = 864 | 26.13 (± 4.77) Range: (5.0 ; 37.9) N = 81 | **0.032** |
| **Clinical history of diabetes** Diabetes with organ disfunction Diabetes without organ disfunction Diabetes without organ dysfunction No No diabetes Yes without organ dysfunction | 29 (1.61%) 257 (14.28%) 2 (0.11%) 20 (1.11%) 1488 (82.67%) 4 (0.22%) N = 1800 | 1 (0.83%) 22 (18.33%) 0 (0.0%) 0 (0.0%) 97 (80.83%) 0 (0.0%) N = 120 | 0.676 |
| **Clinical history of chronic pulmonary disease (other than Covid-19 pneumonia)** Yes No | 159 (8.83%) 1641 (91.17%) N = 1800 | 7 (5.83%) 113 (94.17%) N = 120 | 0.335 |
| **Clinical history of hypertension** Yes No | 714 (39.67%) 1086 (60.33%) N = 1800 | 44 (36.67%) 76 (63.33%) N = 120 | 0.579 |
| **Clinical history of atrial fibrillation** Yes No | 115 (6.39%) 1685 (93.61%) N = 1800 | 7 (5.83%) 113 (94.17%) N = 120 | >0.999 |
| **Clinical history of ischemic heart disease** Yes No | 147 (8.17%) 1652 (91.83%) N = 1799 | 7 (5.83%) 113 (94.17%) N = 120 | 0.46 |
| **Clinical history of chronic kidney disease** No  Yes - in permanent renal replacement therapy  Yes - under medications | 1742 (96.78%) 11 (0.61%) 47 (2.61%) N = 1800 | 113 (94.17%) 1 (0.83%) 6 (5.0%) N = 120 | 0.172 |
| **Clinical history of diseases of the hematopoietic system** Yes  No | 39 (2.17%) 1761 (97.83%) N = 1800 | 2 (1.67%) 118 (98.33%) N = 120 | >0.999 |
| **Patient on immunosuppressive medications on hospital admission** Yes No | 37 (2.06%) 1763 (97.94%) N = 1800 | 1 (0.83%) 119 (99.17%) N = 120 | 0.511 |
| **Glasgow coma scale (GCS). Available at: https://www.mdcalc.com/glasgow-coma-scale-score-gcs** | 14.98 (± 0.156) Range: (13.0 ; 15.0) N = 1103 | 14.98 (± 0.224) Range: (13.0 ; 15.0) N = 80 | 0.943 |
| **qSOFA score. Available at: https://www.mdcalc.com/qsofa-quick-sofa-score-sepsis)** 0 1 2 3 | 715 (89.15%) 63 (7.86%) 16 (2.0%) 8 (1.0%) N = 802 | 56 (93.33%) 3 (5.0%) 1 (1.67%) 0 (0.0%) N = 60 | 0.919 |
| **WSES sepsis score. Available at: https://wjes.biomedcentral.com/articles/10.1186/s13017-015-0055-0/tables/5** 0 1 2 3 4 5 | 391 (58.53%) 21 (3.14%) 196 (29.34%) 48 (7.19%) 2 (0.3%) 10 (1.5%) N = 668 | 34 (65.38%) 2 (3.85%) 10 (19.23%) 6 (11.54%) 0 (0.0%) 0 (0.0%) N = 52 | 0.451 |
| **BISAP (Bedside Index of Severity in Acute Pancreatitis) score. Available at: https://www.mdcalc.com/bisap-score-pancreatitis-mortality** 0 1 2 3 4 | 302 (41.2%) 314 (42.84%) 98 (13.37%) 18 (2.46%) 1 (0.14%) N = 733 | 33 (55.93%) 17 (28.81%) 7 (11.86%) 2 (3.39%) 0 (0.0%) N = 59 | 0.156 |
| **ASA score** 1 2  3 4 | 292 (27.68%) 478 (45.31%) 260 (24.64%) 25 (2.37%) N = 1055 | 20 (22.99%) 39 (44.83%) 22 (25.29%) 6 (6.9%) N = 87 | 0.081 |
| **Glasgow-Imrie criteria for severity of acute pancreatitis. Available at: https://www.mdcalc.com/glasgow-imrie-criteria-severity-acute-pancreatitis**  0 1 2 3 4 5 | 195 (26.82%) 308 (42.37%) 153 (21.05%) 53 (7.29%) 12 (1.65%) 6 (0.83%) N = 727 | 20 (37.74%) 19 (35.85%) 9 (16.98%) 5 (9.43%) 0 (0.0%) 0 (0.0%) N = 53 | 0.55 |
| **Ranson's criteria for pancreatitis mortality. Available at: https://www.mdcalc.com/ransons-criteria-pancreatitis-mortality** 0 1 2 3 4 5 | 140 (19.77%) 266 (37.57%) 181 (25.56%) 89 (12.57%) 25 (3.53%) 7 (0.99%) N = 708 | 22 (39.29%) 13 (23.21%) 14 (25.0%) 2 (3.57%) 4 (7.14%) 1 (1.79%) N = 56 | **0.003** |
| **APACHE II score** | 5.94 (± 3.35) Range: (0.0 ; 18.0) N = 575 | 6.64 (± 3.96) Range: (0.0 ; 18.0) N = 50 | 0.198 |
| **Temperature on admission (°C)** | 36.7 (± 2.17) Range: (26.0 ; 123.0) N = 1800 | 67.12 (± 334.88) Range: (34.3 ; 3705.0) N = 120 | 0.468 |
| **Systolic blood pressure on admission (mmHg)** | 133.48 (± 20.76) Range: (70.0 ; 212.0) N = 1800 | 133.32 (± 21.13) Range: (70.0 ; 194.0) N = 120 | 0.92 |
| **Heart rate on admission (bpm)** | 78.81 (± 15.2) Range: (32.0 ; 178.0) N = 1799 | 79.08 (± 12.72) Range: (54.0 ; 130.0) N = 119 | 0.711 |
| **Respiratory rate on admission (number of breaths/min)** | 16.12 (± 4.4) Range: (10.0 ; 99.0) N = 1777 | 15.81 (± 3.56) Range: (10.0 ; 30.0) N = 110 | 0.439 |
| **Blood oxygen saturation level on admission (SpO2%)** | 97.46 (± 1.95) Range: (75.0 ; 100.0) N = 1796 | 97.44 (± 1.73) Range: (93.0 ; 100.0) N = 117 | 0.673 |
| **WBC on admission (cells/mm3)** | 11.77 (± 4.6) Range: (2.3 ; 37.8) N = 1559 | 12.87 (± 5.34) Range: (3.9 ; 37.7) N = 120 | **0.047** |
| **Neutrophils on admission (cells/mm3)** | 9.46 (± 4.43) Range: (1.2 ; 35.9) N = 1381 | 10.22 (± 4.49) Range: (2.2 ; 22.9) N = 117 | 0.105 |
| **Platelets on admission (mcL)** | 248.44 (± 82.56) Range: (37.0 ; 753.0) N = 1538 | 249.68 (± 78.4) Range: (85.0 ; 610.0) N = 120 | 0.849 |
| **INR - International Normalized Ratio on admission** | 1.13 (± 0.387) Range: (0.47 ; 5.76) N = 1332 | 1.15 (± 0.384) Range: (0.75 ; 3.74) N = 110 | 0.158 |
| **C-Reactive Protein on admission (mg/L)** | 59.12 (± 65.06) Range: (-7.0 ; 450.0) N = 1319 | 57.86 (± 61.34) Range: (2.0 ; 295.0) N = 90 | 0.814 |
| **Aspartate aminotransferase-AST on admission (U/L)** | 203.47 (± 188.55) Range: (7.0 ; 993.0) N = 1236 | 205.38 (± 188.53) Range: (16.0 ; 965.0) N = 103 | 0.699 |
| **Alanine aminotransferase-ALT on admission (U/L)** | 237.27 (± 234.62) Range: (5.0 ; 1847.0) N = 1529 | 250.75 (± 232.21) Range: (11.0 ; 1136.0) N = 120 | 0.254 |
| **Total bilirubin on admission (mg/dL)** | 2.5 (± 2.35) Range: (0.1 ; 21.0) N = 1519 | 2.37 (± 2.26) Range: (0.29 ; 13.4) N = 118 | 0.495 |
| **Conjugated bilirubin on admission (mg/dL)** | 1.56 (± 1.61) Range: (0.0 ; 9.5) N = 943 | 1.51 (± 1.72) Range: (0.1 ; 8.3) N = 64 | 0.368 |
| **Gamma-glutamyl transpeptidase-GGT on admission (U/L)** | 309.64 (± 314.66) Range: (1.0 ; 1887.0) N = 876 | 349.59 (± 421.74) Range: (8.0 ; 2514.0) N = 74 | 0.797 |
| **Serum amylase on admission (U/L)** | 1819.78 (± 2438.33) Range: (300.0 ; 35740.0) N = 1090 | 2136.92 (± 2222.04) Range: (310.0 ; 14000.0) N = 88 | **0.023** |
| **Serum lipase on admission (U/L)** | 5413.74 (± 9062.37) Range: (102.0 ; 73355.0) N = 1165 | 6887.53 (± 10443.67) Range: (164.0 ; 56888.0) N = 91 | 0.122 |
| **Lactate DeHydrogenase-LDH on admission (U/L)** | 353.79 (± 390.4) Range: (1.06 ; 9186.0) N = 780 | 303.11 (± 185.72) Range: (110.0 ; 1284.0) N = 76 | 0.131 |
| **Procalcitonin on admission (ng/mL)** | 1.23 (± 4.26) Range: (0.0 ; 45.0) N = 243 | 0.304 (± 0.273) Range: (0.03 ; 1.1) N = 28 | 0.387 |
| **Lactate on admission (mmol/L)** | 1.6 (± 0.954) Range: (0.1 ; 9.17) N = 551 | 1.84 (± 0.99) Range: (0.5 ; 5.6) N = 64 | **0.015** |
| **Initial diagnostic imaging** CT scan 24-48 from hospital admission CT scan 24-48h from admission CT scan <24 hours from hospital admission CT scan >48 hours from hospital admission  CT scan on admission MRCP Ultrasound scan on admission | 49 (2.72%) 1 (0.06%) 57 (3.17%) 47 (2.61%) 331 (18.39%) 40 (2.22%) 1275 (70.83%) N = 1800 | 2 (1.67%) 0 (0.0%) 3 (2.5%) 2 (1.67%) 20 (16.67%) 2 (1.67%) 91 (75.83%) N = 120 | 0.972 |
| **MRCP** Yes No | 573 (31.83%) 1227 (68.17%) N = 1800 | 48 (40.0%) 72 (60.0%) N = 120 | 0.08 |
| **Endoscopic ultrasound scan -EUS** No Yes, Endoscopic Ultrasound Scan <24 hours from admission Yes, Endoscopic Ultrasound Scan >24 hours from admission | 1572 (87.33%) 31 (1.72%) 197 (10.94%) N = 1800 | 108 (90.0%) 2 (1.67%) 10 (8.33%) N = 120 | 0.669 |
| **Re-evaluation with CT scan** <14 days > 14 days | 397 (87.64%) 56 (12.36%) N = 453 | 20 (83.33%) 4 (16.67%) N = 24 | 0.526 |
| **Abdominal findings** Diffuse abdominal pain Diffuse abdominal rigidity Localized abdominal pain Localized abdominal rigidity No abdominal pain/No abdominal rigidity | 277 (15.39%) 13 (0.72%) 1377 (76.5%) 106 (5.89%) 27 (1.5%) N = 1800 | 28 (23.33%) 1 (0.83%) 86 (71.67%) 4 (3.33%) 1 (0.83%) N = 120 | 0.167 |
| **Choledocholithiasis** No Yes Yes, with common bile duct obstruction | 1431 (79.5%) 270 (15.0%) 99 (5.5%) N = 1800 | 95 (79.17%) 16 (13.33%) 9 (7.5%) N = 120 | 0.603 |
| **Emdoscopic Retrograde Cholangio Pancreatography** No Yes, > 72 hours from hospital admission Yes, within 24 hours from hospital admission Yes, within 24-48 hours from hospital admission Yes, within 48-72 hours from hospital admission | 1501 (83.39%) 116 (6.44%) 22 (1.22%) 54 (3.0%) 107 (5.94%) N = 1800 | 101 (84.17%) 6 (5.0%) 2 (1.67%) 3 (2.5%) 8 (6.67%) N = 120 | 0.945 |
| **Use of somatostatin analogs** Yes No | 110 (6.11%) 1690 (93.89%) N = 1800 | 6 (5.0%) 114 (95.0%) N = 120 | 0.842 |
| **Nutritional support on admission** Enteral via naso-gastric feeding tube Enteral via naso-jejunal feeding tube Nihil per os Oral Total parental nutrition | 9 (0.5%) 3 (0.17%) 749 (41.61%) 911 (50.61%) 128 (7.11%) N = 1800 | 0 (0.0%) 0 (0.0%) 57 (47.5%) 56 (46.67%) 7 (5.83%) N = 120 | 0.726 |
| **Which of the following AP guidelines you have adhered to in the treatment of this patient?** Other The 2005 UK guidelines for the management of Acute Pancreatitis  The 2015 Japanese guidelines for the management of acute pancreatitis  The 2018 AGA Institute Guideline on Initial Management of Acute Pancreatitis  The 2019 WSES guidelines for the management of Acute Pancreatitis patients  The 2020 American Gastroenterological Association Clinical Practice Update | 254 (20.22%) 108 (8.6%) 34 (2.71%) 163 (12.98%) 676 (53.82%) 21 (1.67%) N = 1256 | 26 (28.26%) 6 (6.52%) 0 (0.0%) 18 (19.57%) 40 (43.48%) 2 (2.17%) N = 92 | 0.069 |

**Supplementary materials 3.**

Univariable analysis comparing patients who experience recurrent acute pancreatitis leading to hospital readmission within 30 days. ABP, acute biliary pancreatitis.

Significant comparisons are reported in **bold.**

Continuous data are presented as means with standard deviations; the range is also reported. Categorical data are presented as numbers and percentages.

Under each variable the number of patients with available data is reported.
